# Comparative analysis of freshwater phytoplankton communities in two lakes of Burabay National Park using morphological and molecular approaches

**DOI:** 10.1038/s41598-021-95223-z

**Published:** 2021-08-09

**Authors:** Dmitry V. Malashenkov, Veronika Dashkova, Kymbat Zhakupova, Ivan A. Vorobjev, Natasha S. Barteneva

**Affiliations:** 1 National Laboratory Astana, Nazarbayev University, Nur-Sultan, Kazakhstan; 2 School of Engineering and Digital Sciences, Nazarbayev University, Nur-Sultan, Kazakhstan; 3 Core Facilities, Nazarbayev University, Nur-Sultan, Kazakhstan; 4 Department of Biology, School of Sciences and Humanities, Nazarbayev University, Nur-Sultan, Kazakhstan; 5 EREC, Nazarbayev University, Nur-Sultan, Kazakhstan; 6 Present Address: Department of General Ecology and Hydrobiology, Lomonosov Moscow State University, Moscow, Russian Federation

**Keywords:** Ecology, Ecology

## Abstract

We analyzed phytoplankton assemblages’ variations in oligo-mesotrophic Shchuchie and Burabay lakes using traditional morphological and next-generation sequencing (NGS) approaches. The total phytoplankton biodiversity and abundance estimated by both microscopy and NGS were significantly higher in Lake Burabay than in Lake Shchuchie. NGS of 16S and 18S rRNA amplicons adequately identify phytoplankton taxa only on the genera level, while species composition obtained by microscopic examination was significantly larger. The limitations of NGS analysis could be related to insufficient coverage of freshwater lakes phytoplankton by existing databases, short algal sequences available from current instrumentation, and high homology of chloroplast genes in eukaryotic cells. However, utilization of NGS, together with microscopy allowed us to perform a complete taxonomic characterization of phytoplankton lake communities including picocyanobacteria, often overlooked by traditional microscopy. We demonstrate the high potential of an integrated morphological and molecular approach in understanding the processes of organization in aquatic ecosystem assemblages.

## Introduction

Natural lake systems represent essential reservoirs for domestic water supply, fish production, and recreational activities. At the same time, however, lakes are among the most vulnerable ecological systems and, therefore, should be continuously monitored^1–4^. Growing drainage shortage and diffuse source pollution of natural water reservoirs have severely impacted the water resources of Kazakhstan—the largest nation in Central Asia and one of the most water-scarce countries on the Eurasian continent^5^. Anthropogenic pressure and weather fluctuations resulting in alterations of physico-chemical conditions, nutrient input, eutrophication, and increase of grazing pressure, among other issues, cause substantial changes in the structure and functioning of lake ecosystems^4,6–8^. The observed temperature has risen twice as fast in Central Asian countries since the 1970s in comparison with the average global level^9^. Phytoplankton communities respond rapidly to shifting environmental conditions^10^ via changes in cell abundance, morphology, and biomass^11–13^. Phytoplankton development results from interactions between internal processes and external environmental biotic and abiotic factors^14^, including between others temperature, pH and salinity^15–17^. However, biological monitoring methods can provide more insight into the effect of changes in the abiotic chemical and physical parameters of the organisms^18^. The presence of certain taxonomic phytoplankton groups may be used as indicators of chemical and/or physical conditions of the surrounding environment, or water quality^19,20^.

Traditional approaches assessing phytoplankton diversity, distribution, and abundance of phytoplankton taxa, based on morphological characteristics obtained by light microscopy^21–24^ have a number of limitations: (1) labor intensity that limits the size of the quantified sample to hundred(s) of cellular events and a relatively low number of samples to be processed; (2) accurate diagnostics of taxa and their abundances are hampered by undifferentiated morphologies, unidentified early-life algal stages and numerous cryptic species^25,26^; and (3) incomplete description of the changes in biodiversity based on a limited number of morphologically identified taxa. During the last two decades, cytometric methods (flow cytometry (FCM) and imaging flow cytometry (IFC)) have been recognized as a powerful tool to study seasonal and spatial trends of phytoplankton^27,28^. It is noteworthy, however, that conventional cytometry may not be allowed to isolate and characterize all plankton species and colonial forms identified by traditional microscopy due to size limitations of flow cells typically within a 150 μm limit^29^.

Molecular monitoring tools represent a promising alternative to morphological methods. Moreover, after the unveiling of the key role of picoplankton in aquatic food webs and primary production^30^, a demand arose for new precision and sensitive techniques for the detection and characterization of these microorganisms. Many of them are too small to be identified by light microscopy. Therefore, molecular methods became one of the main tools for defining the composition of picoplankton assemblages^31–35^ as well as for discriminating from harmful planktonic microalgae^36–38^. These tools came into use through biodiversity studies of algae expanding our discovery of new microalgae that were not detected by microscopy^39,40^. New genome data has provided many novel insights into the evolutionary history of photoautotrophic microorganisms^41^. Although next-generation sequencing allows for faster analysis^42^, the identification of unique and fragile nano- and picophytoplankton, the discovery of hidden diverse new microorganisms^43,44^, and minimizes the role of subjective evaluation, comparative studies with traditional methods are still needed^40,42^. Recently, the progress in next-generation sequencing (NGS) facilitated the extensive sequence-based characterization of diverse plankton communities^34,43,45,46^. However, despite the fast development of high-throughput sequencing (HTS), it is not yet clear to what extent the results of a traditional light microscopy-based taxonomic approach can be consistent in comparison with the results of NGS analysis^42^. Comparative methodological studies are also required to evaluate different algal taxa, such as diatoms and cyanobacteria^40,44,47–51^.

In the present study, we compared the effectiveness of optical methods and NGS in assessing two Kazakhstani lakes located in the Burabay National park (Lake Burabay and Lake Shchuchie).

## Methods

### Sampling sites description

**Figure 1:**
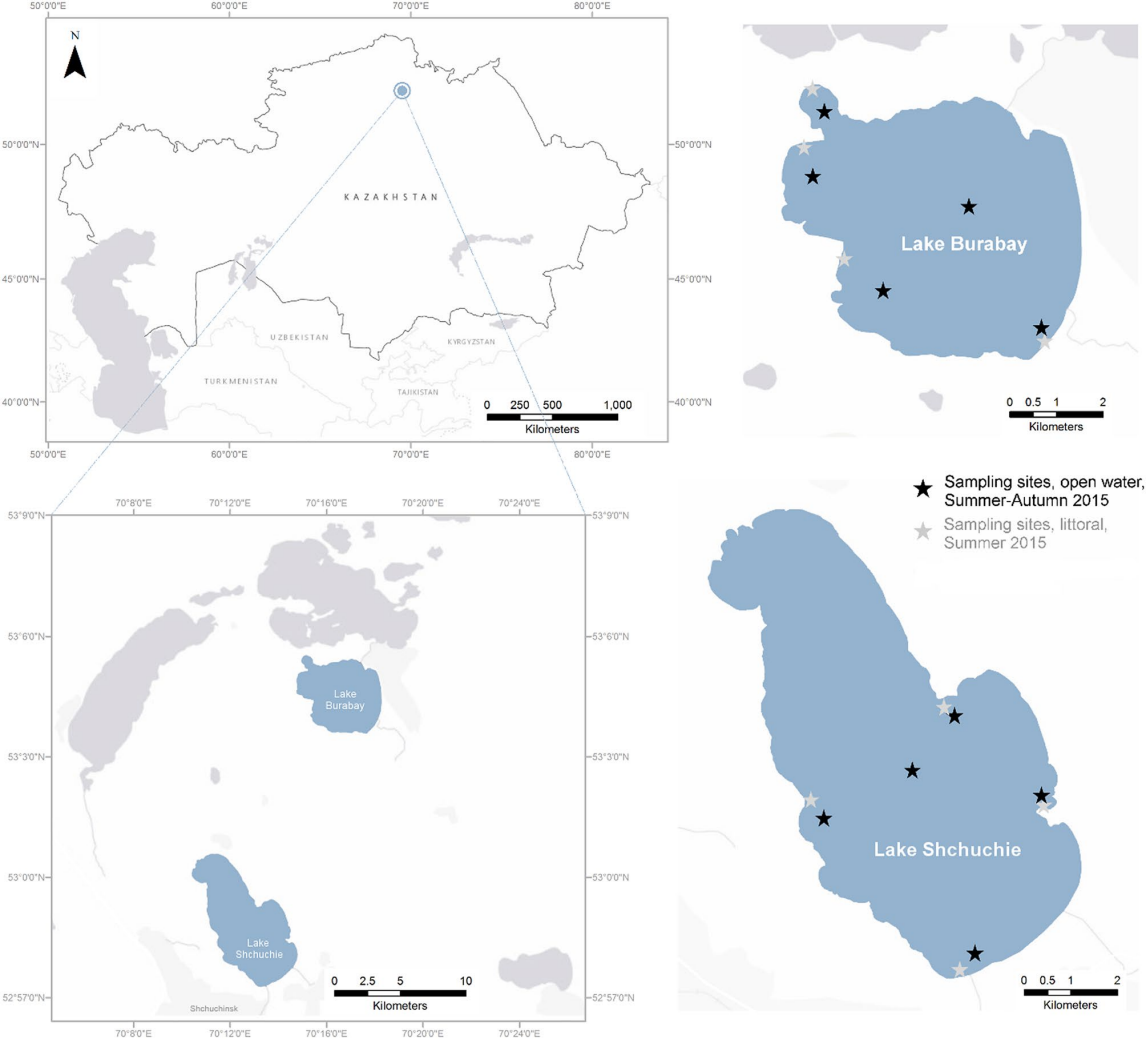
Geographical location of sampling sites at Lakes Burabay and Shchuchie in Burabay National Nature Park in 2015. The figure was created using ArcGis Pro 2.7.0 (Esri Inc., USA) software (https://www.arcgis.com/index.html).

Burabay National Park is located in the northern part of Kazakhstan (the Akmola region) and includes fourteen lakes, of which Burabay and Shchuchie are among the largest (Fig. 1). This region is characterized by a continental climate, with warm summers (the average temperature in July is + 18.0 to + 20.5 °C), cold winters (the average temperature in January is − 16.0 to − 19.0 °C), and an average annual rainfall of 250 to 350 mm^52^. The beds of lakes Burabay and Shchuchie are of tectonic origin, filled with freshwater where bicarbonate, sulfate, and calcium ions prevail (Suppl. Table S1). Lake Burabay (Auliekol’, Borovoe) is an oligo-mesotrophic lake that may be defined as a continuous cold polymictic lake (Osgood Index = 1.05)^53,54^. It is a shallow lake (mean depth = 3.4 m, mean Secchi depth = 2.8 m) (Suppl. Tables S1, S2), with the bottom largely (up to 40–70%) covered by submerged macrophytes, both angiosperms (particularly *Potamogeton* species and *Ceratophyllum demersum*) and stoneworts (*Chara* species). As the main recreational area in the region that includes beach activities, swimming, boating, and fishing, it also has several hotels in its surrounding area. Thus, the ecosystem of Lake Burabay is under a strong recreational load. Lake Shchuchie (Shortan, Shortandy, Shortankol’) is an oligotrophic closed lake. Although its maximum depth reaches 22.7 m^55^ (Suppl. Table S2), the lake has no continuous stratification of a water column (possibly due to subaquatic springs) and may be classified as a cold polymictic lake (Osgood Index = 2.9)^53^. The ecological state of Lake Shchuchje is intensively influenced by the City of Shchuchinsk on the north shore. Water from the lake is used for various purposes-drinking, domestic use, and industrial use^56,57^.

### Field sampling and data collection

Surface water sampling was carried out monthly from June to September in 2015 from Lakes Burabay and Shchuchie (Fig. 1). Sampling locations were chosen based on morphometric characteristics of the lakes and heterogeneity of the degree of anthropogenic load. Samples for quantitative microscopy analyses of the phytoplankton community were collected from the surface water horizon (0.5 m depth) at each location using 0.5–1.0 L plastic containers. Modified Lugol’s iodine solution with the addition of formaldehyde and glacial acetic acid was used for longer preservation of the sample^58^. Collected quantitative samples were concentrated by a settling method^59^. Water samples for qualitative analyses were collected in duplicates using an Apstein plankton net^60^. The two sample replicates consisted of live unfixed material that was analyzed directly on arrival to the laboratory, and samples fixed with glutaraldehyde at the final concentration of 2% for identification of fragile phytoflagellates^61^. Water samples for cytometry analyses were collected and analyzed alive or fixed with 0.5% glutaraldehyde at final concentration until the analysis. Samples for molecular analysis (1.5–2 L) were filtered through 0.2 µm pore size polyethersulfone filters (EMD-Millipore, USA), placed into 5 mL bead tubes provided in the PowerWater DNA isolation kit (MO BIO Inc., USA), and stored at − 20 °C until DNA extraction.

Physico-chemical parameters such as water temperature, pH, dissolved oxygen (DO), conductivity, total dissolved solids (TDS) were measured at each sampling point using a YSI Pro Plus multimeter (Xylem Inc., UK) simultaneously with phytoplankton samples. Water transparency (Secchi depth) was dimensioned using a black and white Secchi disk (diameter 0.20 m). Water samples were filtered through 0.22 µm polycarbonate filters using a vacuum pump, and the filtrate was used for subsequent ion chromatography (IC) analysis. Measurements of concentrations of nitrates (NO_3_^–^), nitrites (NO_2_^–^), ammonium (NH_4_^+^), phosphates (PO_4_^3–^), fluorides (F^–^), chlorides (Cl^–^), bromides (Br^–^), sulfates (SO_4_^2–^), lithium (Li^+^), calcium (Ca^2+^), sodium (Na^+^), potassium (K^+^), magnesium (Mg^2+^) were performed using Compact IC FLEX 930 with titrator Titrando 905 Metrohm (Mettler-Toledo, Switzerland) (Suppl. Table S1).

### Phytoplankton identification and counting using microscopy

Identification and quantification of phytoplankton cells were performed under Leica DM500 (Leica Microsystems, Germany) and Nikon Eclipse E200F (Nikon Instruments Inc., USA) microscopes equipped with phase contrast. Phytoplankton cells were counted using Palmer-Maloney-type 0.05 mL counting slide^62^ with Nageotte-type grid on its bottom under working magnification of × 400. The number of cells calculated for each sample was not less than 3000 cells^58^. The × 63 and × 100 objectives were used for the identification of live and fixed phytoplankton cells and colonies. Calculation of biovolumes of phytoplankton cells was based on geometric assignation^63–67^. LAS EZ software (Leica Microsystems, Germany) and NIS-Elements (Nikon Instruments Inc., USA) were used for sizing of linear dimensions of cells. Total phytoplankton biovolume was taken as the sum of biovolumes of all phytoplankton cells and converted into biomass in terms of µg/mL.

### Flow cytometry and cell sorting

Flow cytometry analysis and cell sorting were performed using a 6-laser SORP FACSAria equipped with a combination of 355, 405, 488, 561, 594, and 640 nm lasers (BD Biosciences, USA). Daily calibration of the flow cytometer was performed using 6.0 μ Alignflow beads (Life Technologies, USA) and 6-peaks 3.0 μ Rainbow calibration particles (Spherotech, USA). Phytoplankton subpopulations were discriminated based on autofluorescence collected using 620/20 (APC), 575/25 (PE), 695/40 (PerCP) bandpass filters. At least 15,000 events from selected populations were sorted at 20 psi using a 100 µ nozzle.

### FlowCam analysis

An imaging flow cytometer FlowCam VS-4 (Yokagawa Fluid Imaging Technologies, USA) was used to analyze phytoplankton samples as described early^68^. Calibration of the instrument was performed using a mixture of 5 µm, 10 µm, and 25 µm size Focus beads (Yokagawa Fluid Imaging Technologies, USA). Live samples were run in laser-triggered mode using a 10× objective and a 100 µL flow cell at a flow rate of 0.15 mL/min for 10–20 min. Images were recorded at a rate of 20 frames per second and were analyzed using VisualSpreadSheet software vs. 4.0 (Yokagawa Fluid Imaging Technologies, USA).

### Next-generation sequencing

Genomic DNA was extracted using PowerWater DNA Isolation Kit (MO BIO Laboratories, Inc, USA) according to the manufacturer’s instructions with extended lysis time (15 min at 65 °C). The final elution of DNA was performed with 50 μL 10 mM Tris (MO BIO buffer PW6). DNA concentrations were quantified using a Qubit instrument (Life Technologies Inc., USA) with a double-stranded DNA specific dye (dsDNA BR assay, Life Technologies Inc., USA). Samples with DNA yields less than 10 ng/μL were processed through standard ethanol precipitation to increase DNA concentration. Ethanol-precipitated pellets were re-suspended with 25 μL 10 mM Tris (MO BIO buffer PW6).

Polymerase chain reaction (PCR) amplified the hypervariable V4 region of 16S (341F (5′-CGGTAAYTCCAGCTCYV-3′)/805R (5′-GACTACHVGGGTATCTAATCC-3′)) and 18S (574F (5'-GCG GTA ATT CCA GCT CCA A-3′)/1132R (5′-ACG GCC ATG CAC CAC CAC CCA T-3′)) rRNA gene sequences using Promega PCR master mix (Promega, USA).

The PCR reaction mixture consisted of 12.5 μL master mix, 2.5 μL primer F, 2.5 μL primer R, 5 μL DNA, 2.5 μL H_2_O. PCR conditions for prokaryotic primers 341F&805R consisted of an initial denaturation step of 5 min at 95 °C followed by 40 cycles of 40 s at 95 °C, 40 s at 53 °C, 1 min at 72 °C, and a final elongation step of 7 min at 72 °C. For eukaryotic primers 574*F&1132R: an initial denaturation step of 5 min at 95 °C followed by 25 cycles of 1 min at 98 °C, 20 s at 98 °C, 20 s at 51 °C, 12 s at 72 °C, and a final elongation step, 1 min at 72 °C. PCR products were verified using 1% agarose gel electrophoresis.

Sequencing was performed on the Illumina MiSeq platform (Illumina Inc., USA) at Fasteris SA (Switzerland). Base calling was conducted using MiSeq Control Software (MCS) vs.2.4.1.3, RTA1.18.54.0, and CASAVA-1.8.2 pipelines. The Trimmomatic package vs. 0.32 was used for sequences trimming^69^. The quality scores associated with each base call for each read were used to determine the portion of each Illumina read that was of acceptable quality. Two paired-end reads were joined on the overlapping ends using the fastq-join tool from the ea-utils package vs.1.1.2-537^70^. A minimum of 6 bases with up to 8% of mismatches was allowed between each end.

The alignment of sequences was done using the mapping software BWA^71^ against Greengenes (gg_otus_4feb2011 (downloaded in March 2021)) and SILVA (SSURef_NR99_115_tax_silva_DNA.fasta) databases. The sequences were assigned to operational taxonomic units (OTUs) at 97% similarity. The samtools vs.1.2 (http://www.htslib.org/) were used to compute the number of reads mapped onto each OTU.

### Data processing and statistical analysis

Mann–Whitney rank sum non-parametric test was used to determine significant differences among species distribution analyzed by NGS and microscopy in Lakes Burabay and Shchuchie. The direct comparison of microscopic data with NGS (species & genera distribution) was made from both lakes using Spearman correlation analysis (SigmaPlot, SyStat Software, USA). Linear regression and curve estimation were also performed with this software package. Graphic displays were performed using Microsoft Excel 2010 (Microsoft Corporation), GraphPad Prism 7 (GraphPad Inc., USA), ArcGIS vs. Pro 2.7.0 (Esri Inc, USA), Adobe Photoshop CC2 (Adobe Systems Inc., USA), GIMP vs. 2.8.22.

## Results

### Morphological diversity of phytoplankton based on microscopy and FlowCam analysis

**Figure 2:**
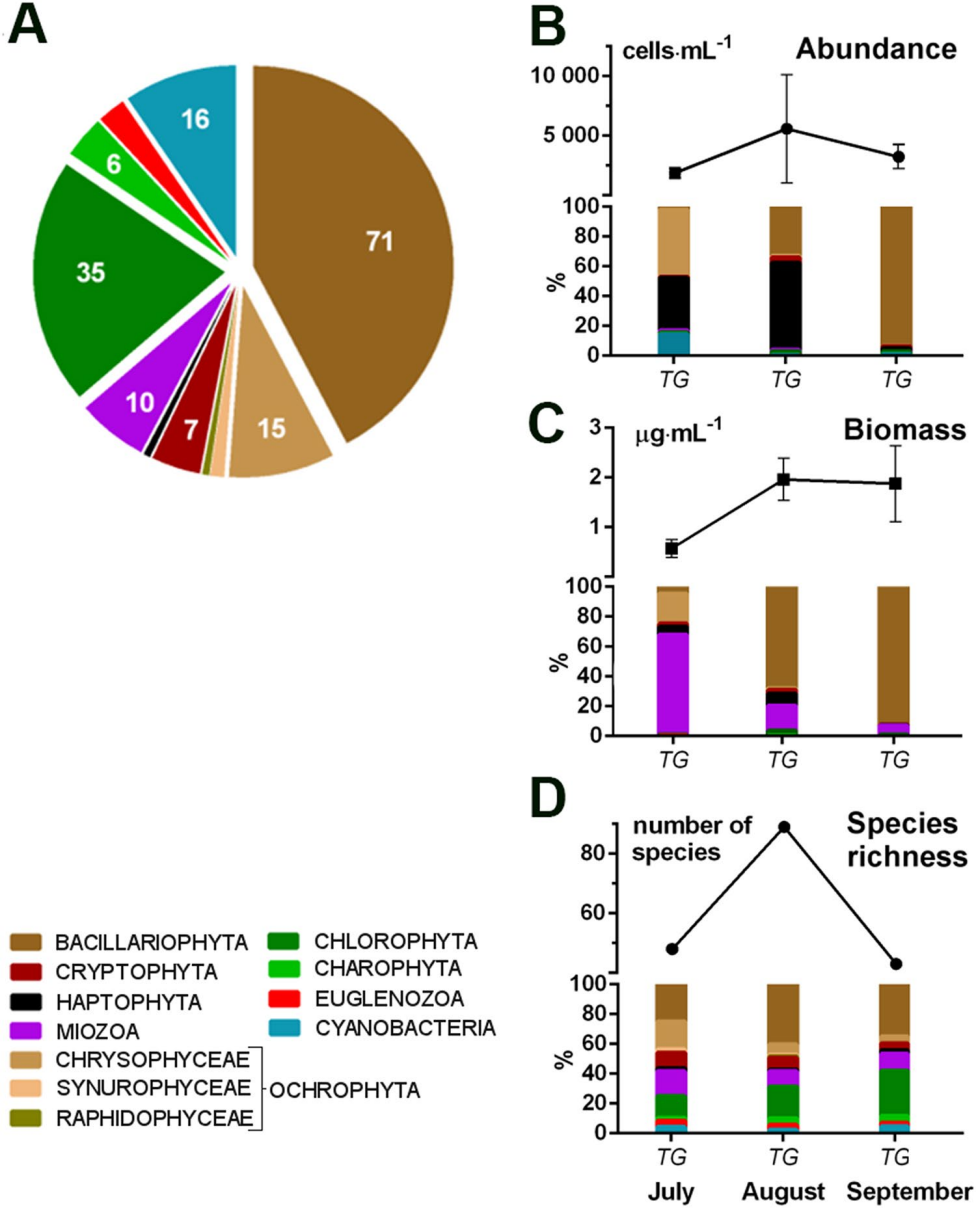
Community composition of major taxonomic groups of phytoplankton in Lake Shchuchie defined by light microscopy. (**A**) Contribution of identified species belonging to major taxonomic groups; (**B**) total and relative abundance of taxonomic groups; (**C**) total and relative biomass of taxonomic groups; (**D**) total and relative species’ number per taxonomic groups. Mean values ± Std Dev.

The phytoplankton community in Lake Shchuchie was represented by nine phyla, with a total number of 167 species observed (Suppl. Table S3). Among all the species, diatoms (Bacillariophyta) formed the most species-rich group, consisting of up to 71 species, whereas chlorophytes, ochrophytes (specifically chrysophytes), cyanobacteria, and dinoflagellates (Miozoa) counted up to 35, 18, 16, and 10 species, respectively (Fig. 2).

**Figure 3:**
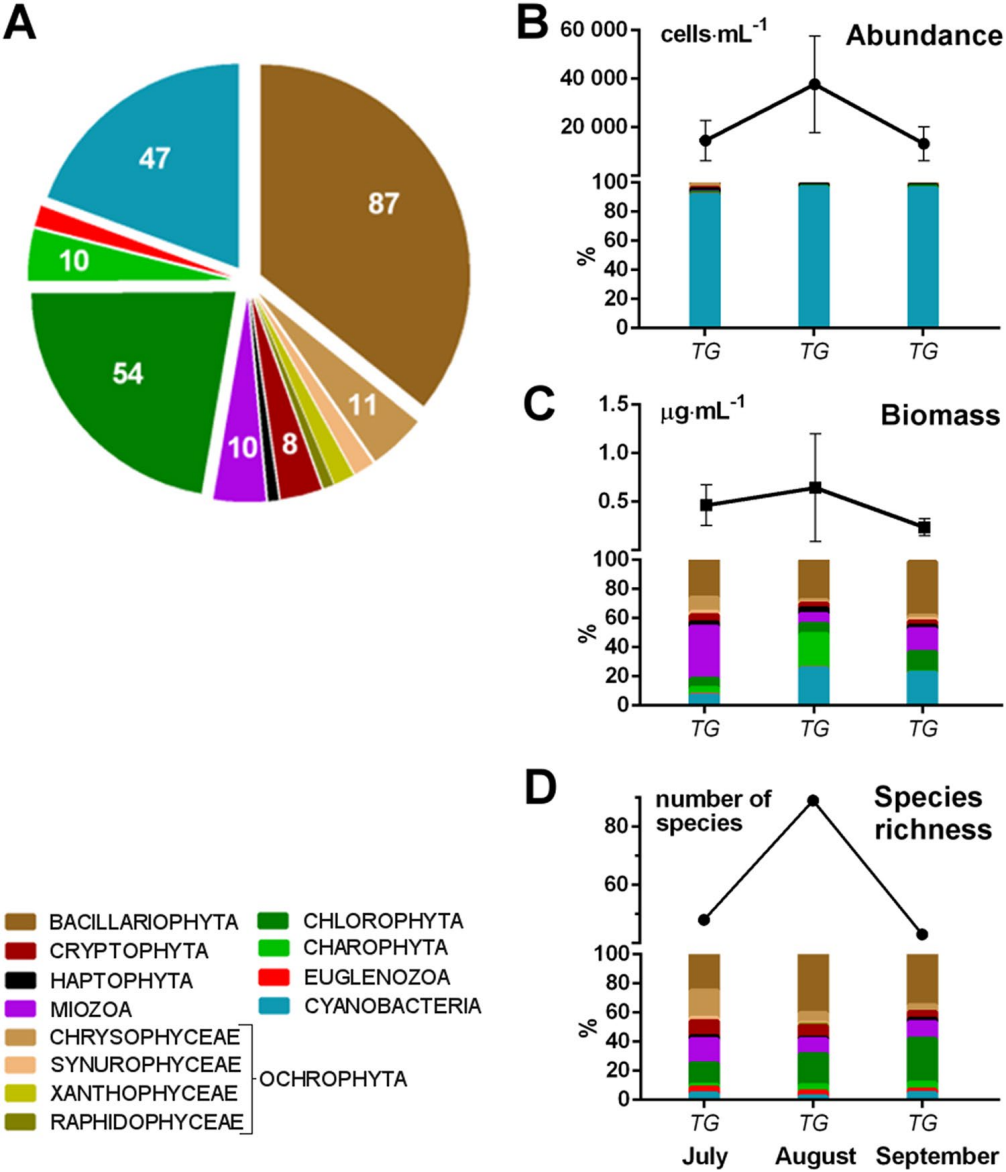
Community composition of phytoplankton in Lake Burabay. (**A**) Contribution of species belonging to major taxonomic groups; (**B**) total and relative abundance of taxonomic groups; (**C**) total and relative biomass of taxonomic groups; (**D**) total and relative species’ number of taxonomic groups. Mean values ± Std Dev.

Both, Lakes Shchuchie and Burabay can be regarded as an oligo-mesotrophic water bodies. Phytoplankton assemblage in Lake Burabay was composed of 243 species from nine phyla, with diatoms (Bacillariophyta) (87 species), chlorophytes (54), and cyanobacteria (47) as the most species-rich groups (Fig. 3, Suppl. Table S3).

The total phytoplankton abundance in Lake Burabay is 6 to 22 times higher than in Lake Shchuchie due to the continual development of small-celled colonial picocyanobacteria (CPCy) typical for shallow nutrient-reach waters^72^ and mainly represented by non-gas-vacuolated *Aphanocapsa*, *Anathece*, and *Cyanodictyon* species (Suppl. Figs. S1, S2). Despite its absolute numerical dominance, the relative biomass of this group is not very significant due to their smaller cell sizes and individual cell biovolumes. Potentially bloom-forming heterocystous cyanoprokaryote *Dolichospermum* (= *Anabaena*) *flos-aquae*, along with *Dolichospermum mucosum*, common for eutrophic lakes with low nitrogen content^72^ were occasionally found at the end of the summer of 2015. The mass development of potentially toxic diazotroph *D.flos-aque*, however, was recorded using FlowCam imaging cytometer in a mid-summer period, when phytoplankton biomass was considerably higher (Suppl. Fig. S3). The highest total abundance and biomass of phytoplankton were observed in August (Fig. 3C,D).

### FACS-based sorting

Monthly collected water samples were also analyzed using light scattering and fluorescence via FACSAria flow cytometer (BD Biosciences, USA). A characteristic flow cytometric “signature” was observed for each lake (Suppl. Fig. S4). In the June samples, from Lake Burabay, it was possible to discriminate three autofluorescent assemblages: one assemblage consisted of small cyanobacteria cells and picoplankton, the second assemblage contained a mixture of *Dinobryon* monads, small cyanobacteria cells, and a few *Cyclotella/Pantocsekiella* spp. cells, and the third assemblage was mostly dominated by *Dinobryon* monads (Suppl. Fig. S4A,B). Later, in the July samples, only two assemblages were observed, each consisting of various cyanobacteria cells mostly dominated by *Cyanodictyon* spp., *Aphanocapsa* spp., and *Anathece* spp. Overall, the sorting results are in agreement with microscopy and FlowCam-based observations, where the majority of phytoplankton cells at the corresponding stations belonged to cyanobacteria. In contrast to Lake Burabay, the samples from Lake Shchuchie analyzed using flow cytometry were more diverse. The major microalgal assemblages from Lake Shchuchie that were discriminated and sorted by the flow cytometer consisted of *Dinobryon* whole cells or its monads, cryptomonads, pico-sized flagellates or picoplankton, and *Cyclotella* spp*.* (Suppl. Fig. S4C,D). Compared to the microscopy analysis, rare phytoplankton groups were missing from the flow cytometry (FCM) analysis with FACSAria instrument, possibly due to their low abundance and/or large size and odd shapes (e.g., long filaments and structures are limited by flow cell size).

### SSU rRNA phytoplankton diversity based on NGS

During next-generation sequencing (NGS) from the MiSeq run of the 16S rRNA and 18S rRNA libraries, raw sequences were acquired from Lake Burabay and Lake Shchuchie sub-samples (at the same 2015 sampling season dates). After applying quality control and clustering procedures, assembled operational taxonomic units (OTUs) were aligned with the Genbank sequence database using a cut-off of 97% sequence identity. To filter only phytoplankton OTUs, a search for keywords was performed, resulting in 114 OTUs (including singletons, 74—without) for 16S rRNA and 369 OTUs for 18S rRNA (including singletons, 228—without). This analysis suggested the dominance (highest read number per OTU) of the uncultured bacteria, *Microcystis*, *Prochlorococcus*, *Synechococcus*, and *Cyanobium* for prokaryotes (16S rRNA sequencing) and *Ceratium hirundinella* for eukaryotic phytoplankton (18S rRNA sequencing). Phylum-level patterns were present, with seasonal variations being largely in agreement with our microscopic and cytometric observations. Subtle patterns identified by microscopy, however, were absent from NGS analysis.

Phytoplankton biodiversity detected by NGS and microscopy were compared at genera and species levels. A morphological approach enabled us to detect species from nine phyla in Lake Shchuchie and Lake Burabay: Bacillariophyta, Ochrophyta (including Chrysophyceae, Synurophyceae, Xanthophyceae, and Raphidophyceae), Miozoa, Haptophyta, Cryptophyta, Chlorophyta, Charophyta, Euglenozoa, and Cyanobacteria. In comparison, the NGS method revealed the same number of phyla, with additional Dictyochophyceae and Eustigmatophyceae classes within the Ochrophyta phylum.

**Figure 4:**
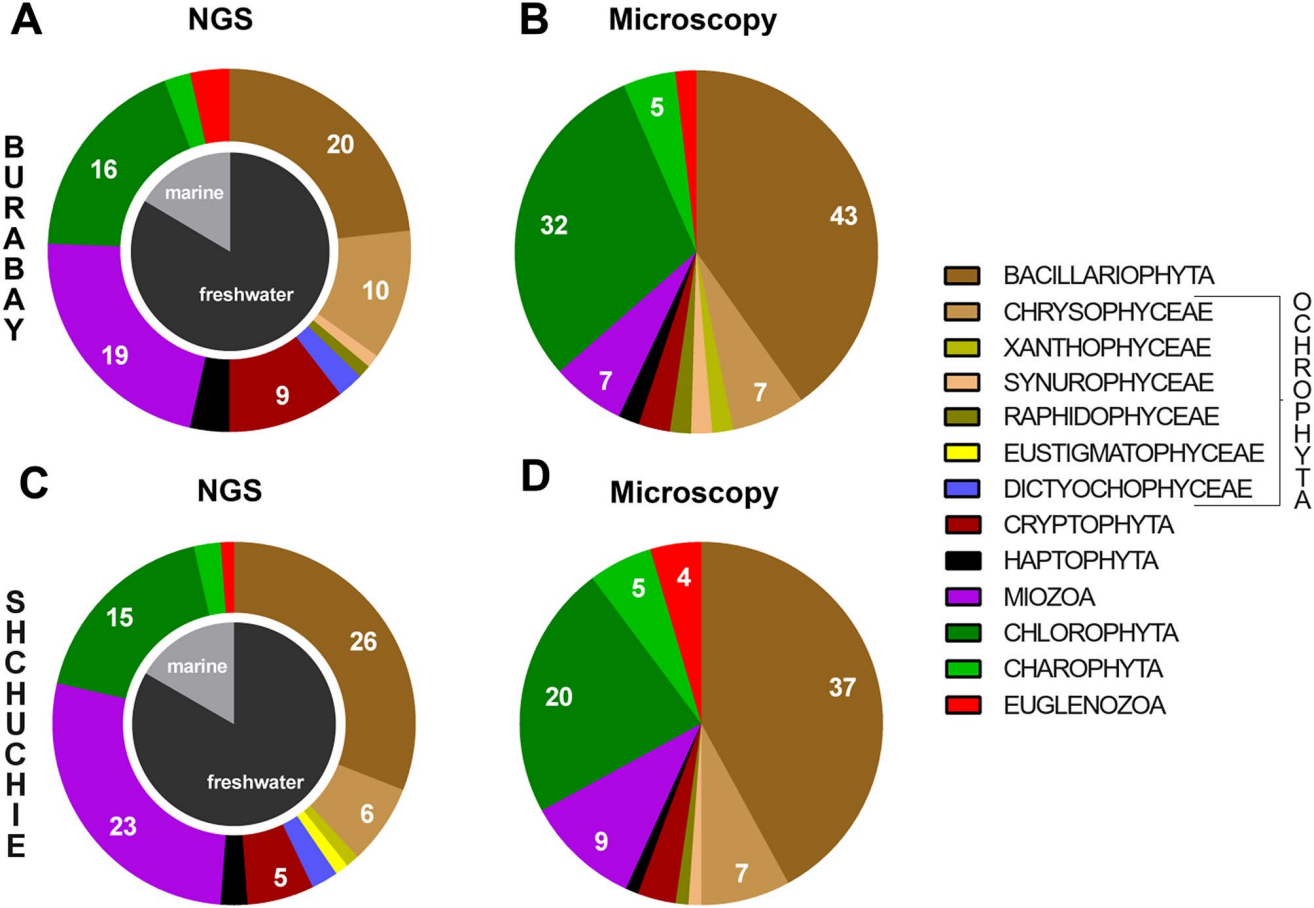
Contribution of different taxonomic groups in genera composition of eukaryotic phytoplankton in Lakes Burabay (**A,B**) and Shchuchie (**C,D**) based on microscopy (**B,D**) and molecular (**A,C**) approaches. Spearman correlative analysis, linear regression and curve estimation (Suppl. File 1) indicated positive correlation between plankton genera distribution defined by microscopic and NGS approaches.

**Figure 5:**
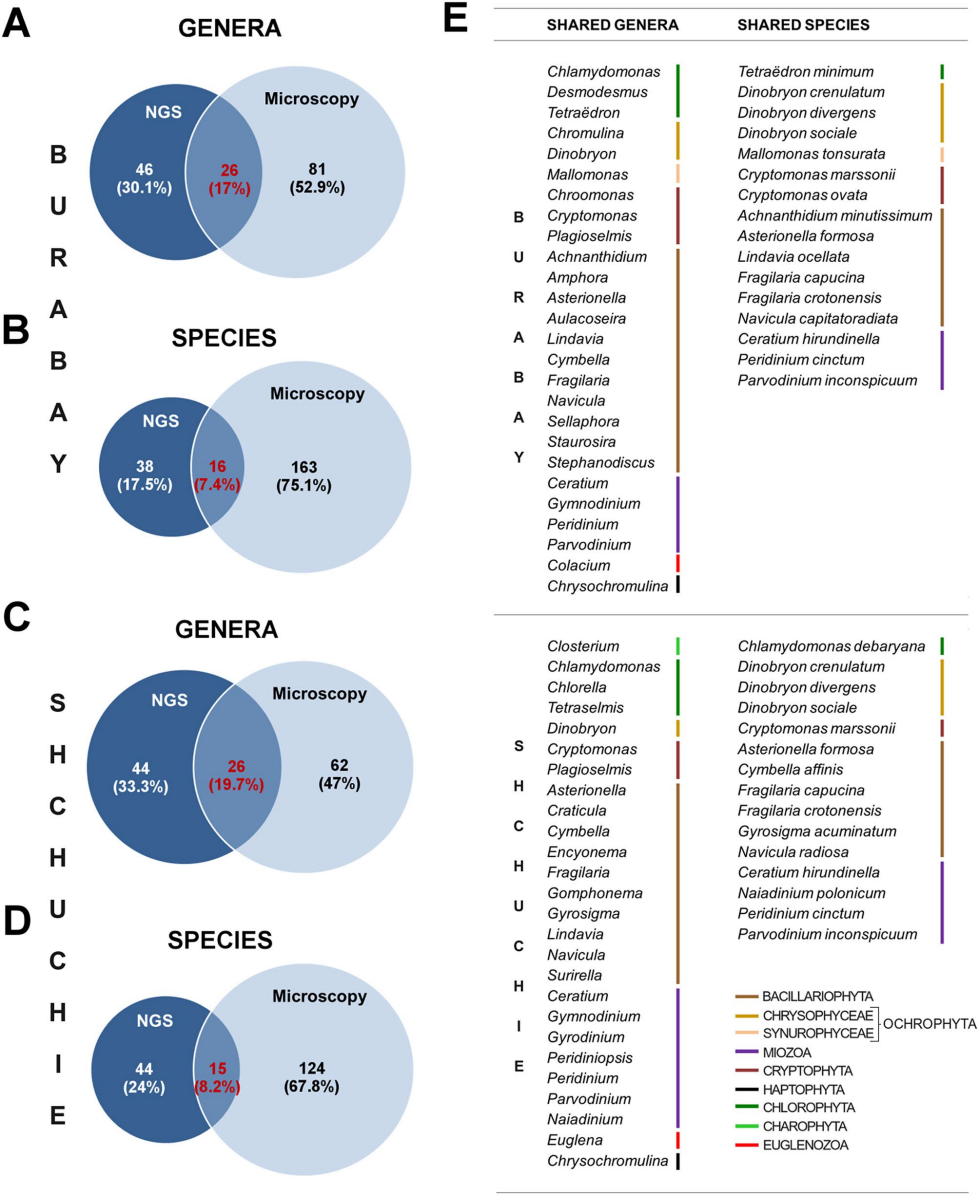
Shared genera and species for eukaryotic phytoplankton of Lakes Burabay and Shchuchie detected by microscopy approach and next-generation sequencing of 18S rRNA gene. Venn diagrams for similar and unique genera ((**A**)—Lake Burabay, (**C**)—Lake Shchuchie) and species ((**B**)—Lake Burabay, (**D**)—Lake Shchuchie) detected by microscopy and NGS. (**E**)—complete list of shared genera and species for phytoplankton of both lakes.

At genera level, almost 17% of all eukaryotic phytoplankton units represent marine phytoplankton genera for both lakes (Fig. 4). For further comparisons between microscopy data and NGS data, only taxonomic units representing freshwater phytoplankton were used. After excluding marine species from our analysis, it was found that the number of genera identified by NGS is less than was identified by microscopy—72 and 70 genera determined by NGS vs. 107 and 88 genera revealed by microscopy for Lake Burabay and Lake Shchuchie, respectively (Figs. 4, 5). A similar situation arose with species-level data when marine and unidentified species were excluded from the analysis (Fig. 5).

The similarity between eukaryotic phytoplankton of the two lakes measured on the basis of data obtained by NGS is higher at the genera level, whereas similarity based on microscopy analysis is higher at the species level. The microscopy survey indicated 66 shared genera and 76 shared species between communities (Jaccard similarity is 0.31 and 0.51, respectively), while NGS detected 42 shared genera and 25 shared species (Jaccard similarity is 0.40 and 0.28, respectively) and thereby found more unique species for each lake.

**Figure 6:**
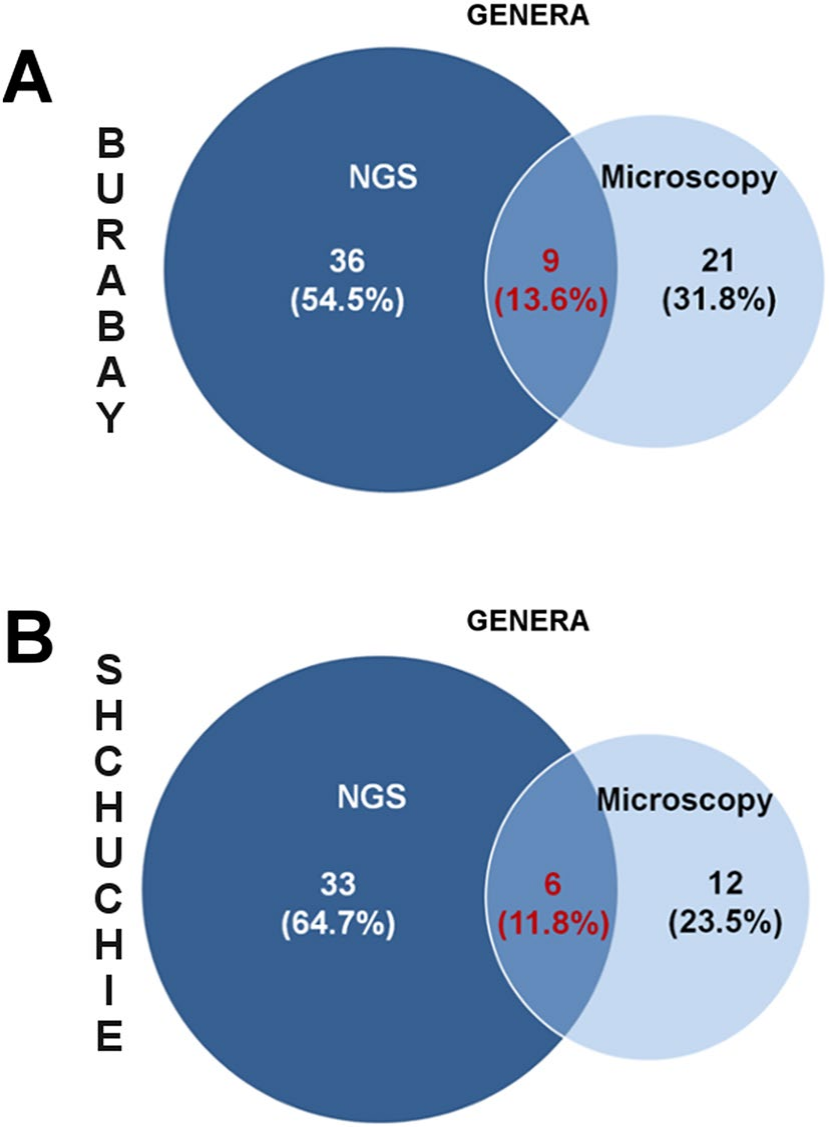
Shared genera for prokaryotic phytoplankton in Lakes Burabay and Shchuchie detected by microscopy approach and next-generation sequencing of 16S rRNA gene. (**A**,**B**) Venn diagrams for similar and unique genera.

Prokaryotic diversity in phytoplankton was higher on genera level based on 16S rRNA analysis than was found through microscopy (Fig. 6). A considerable part of all identified OTUs represents strains so that they cannot be directly compared with the species identified by microscopy. Furthermore, more than half of all identified strains correspond to unicellular picocyanobacteria (PCy) (Suppl. Fig. S5). Interestingly, the majority of these strains are freshwater. Overall, NGS was found to be more sensitive to the detection of planktonic picocyanobacteria in comparison to the light microscopy approach, allowing it to reveal the hidden diversity in freshwater picophytoplankton.

## Discussion

The recent decline in morphological taxonomic studies^73^ makes a quantitative approach invaluable, coupled with traditional analysis by an expert taxonomist. Morphological optical methods are likely to miss the rare (because the size of the sample is limited) or unclassifiable species that contribute to lake diversity and may overestimate the biodiversity and richness of phytoplankton, helping identify different phenotypes and transitional forms as separate species. The extent of taxonomic coverage by reference database is also important^74^. The NGS approach implemented in this paper identified the lack of sequenced freshwater taxa in currently available databases (SILVA, Greengenes etc.). The incompleteness of reference databases is a challenging issue that hampers the identification and assignment of sequences. This results in a large number of OTUs that cannot be taxonomically classified to the species ranks or even stays unclassified depending on the taxonomic group or region of study^75^. In contrast to a morphological approach, the number of genera from Chlorophyta and Bacillariophyta (which are the most diverse eukaryotic groups in phytoplankton in Lakes Shchuchie and Burabay) identified by NGS is 25–50% less (Fig. 4), suggesting that microscopy is a more efficient method for their detection^76^.

On the other hand, NGS allowed us to find more genera and species of dinoflagellates, cryptomonads, and other phytoflagellates (i.a. haptophytes, dictyochophyceans), which were not identified via microscopy. Nevertheless, the number of shared units on species level is more than two times less than on genera level (Fig. 5), which may be related to a lack of information on freshwater phytoplankton in the reference database (SILVA). It was previously shown by Bazin et al*.*^76^ that approaches based on 18S rRNA gene clone libraries using universal primers are biased toward heterotrophic organisms, and a microscopy approach is necessary to reveal the real diversity among phototrophic taxa.

Thus, the taxonomic resolution of the NGS approach (using 16S and 18S rRNA primers) we employed was not able to reliably provide species-level identification. NGS analysis, however, identified phylum-like patterns presented in July–September. It seems that NGS-based taxonomy can be used at the genus level for freshwater phytoplankton communities and may hamper the detection of subtle ecological effects. Further studies employing traditional and NGS approaches in parallel are required to increase the quantity and quality of algal databases, and expand possibilities for the functional analysis of phytoplankton assemblages. Ideally, species should be defined using an integrative approach, including morphology, genetics, behavior, ecology, and geography^77–79^. Currently, most species descriptions rely on phenotype features. However, traditional phenotypically based taxonomy is challenged by molecular findings that provides greater taxonomic resolution than morphology^80–82^.

Notably, the NGS approach is favorable in detecting of picoplankton species/strains, which cannot be found and/or identified by routine microscopy. This is particularly true for picocyanobacteria, especially for unicellular PCy strains due to their small cell sizes and phenotypical plasticity that make them almost indistinguishable morphologically^83^. Nevertheless, the NGS approach gives us less cumulative information about species richness and phytoplankton abundance in comparison to microscopy for the present. Though FCM is another approach to be considered for picocyanobacterial analysis, there are technological limitations that may not allow FCM to isolate all species described by traditional microscopic analysis^84^. We used FCM as an ancillary instrument supporting selective microscopic findings.

## Conclusions

In summary, if we compare optical methods and DNA-based methods, DNA-based analysis may help to analyze samples at different taxonomic levels and discriminate overlooked cryptic and rare species. The advantages of optical methods are relatively low cost of equipment and a direct description of phytoplankton that cannot be replaced by DNA-technologies. It makes light microscopy still a primary method in the study of phytoplankton. However, an integrative approach of both DNA-based and morphological methods has rarely been used, but as demonstrated here may provide deeper insights into the structure of phytoplankton communities, in particular, picophytoplankton. Due to the growing use of new generation-sequencing methods, a larger amount of genomic data can be expected from the phytoplankton research though our knowledge of the phytoplankton metabolome continued to be incomplete. Combined evaluation, results from traditional and modern techniques and monitoring will be the foremost practice in future phytogeographic research.

## Supplementary Information

Supplementary Information.

## Abbreviations

CPCy: Colonial picocyanobacteria
DO: Dissolved oxygen
FCM: Flow cytometry
IFC: Imaging flow cytometry
IC: Ion chromatography
HTS: High-throughput sequencing
MCS: MiSeq control software
NGS: Next-generation sequencing
OTU(s): Operational taxonomic unit(s)
PCy: Picocyanobacteria
PCR: Polymerase chain reaction
rRNA: Ribosomal ribonucleic acid
Std Dev: Standard deviation
TG: Taxonomic group
TDS: Total dissolved solids
