# Supplementary material for: Comparative analysis of freshwater phytoplankton communities in two lakes of Burabay National Park using morphological and molecular approaches
Source: Sci Rep. 2021 Aug 9;11:16130. doi: 10.1038/s41598-021-95223-z (PMC8352915; doi:10.1038/s41598-021-95223-z)
Supplement: Supplementary file 1 — Supplementary Information. [file 41598_2021_95223_MOESM1_ESM.docx]

**Comparative analysis of freshwater phytoplankton communities in two lakes of Burabay National Park using morphological and molecular approaches**

Dmitry V. Malashenkov^§,1,2,#^, Veronika Dashkova^1,3^, Kymbat Zhakupova^4,^, Ivan A. Vorobjev^1,5^, Natasha S. Barteneva^§,1,5,6^

**Supplementary files:** 3 Tables, 5 Figures, and 1 Supplementary file

**Legends to Supplementary Figures:**

**Supplementary Figure 1. FlowCam image library of most common species in Lake Burabay.** Live samples. 10x objective, 100 μm flow cell. (1) *Cyanodictyon planctonicum.* (2-4) *Aphanocapsa holsatica.* (5) *Aphanocapsa elachista*. (6) *Aphanocapsa* cf. *planctonica.* (7) *Microcystis* cf. *smithii*. (8-9) *Snowella litoralis.* (10) *Aphanothece* sp. (11) *Chroococcus* *minimus.* (12) *Ceratium hirundinella*. (13) *Dinobryon sociale*. (14-16) *Coenococcus* cf*. planctonicus* (17-18) *Sphaerocystis* sp. (19-20) *Chlamydomonas* spp. (21-22) *Cryptomonas curvata*. (23-24) *Cryptomonas reflexa*. (25-26) *Plagioselmis nannoplanctica.* (27) *Cryptomonas marssonii*. (28) *Ulnaria ulna*. (29-30) *Mallomonas* sp. (31) *Tetraëdriella jovetii.* (32) *Gymnodinium lacustre.* Scale bar = 20 μm.

**Supplementary Figure 2. FlowCam image library of most common species in Lake Shchuchie.** Live samples. 10x objective, 100 μm flow cell. (1-6) *Gyrodinium helveticum.* (7) *Asterionella formosa*. (8) *Parvodinium* cf*. umbonatum* (9-10) *Peridiniopsis/Parvodinium* spp. (11) *Ceratium hirundinella*. (12) *Fragilaria crotonensis.* (13) *Dinobryon sociale.* (14-17) *Cryptomonas curvata.* (18) *Plagioselmis nannoplanctica.* (19-20) *Cryptomonas* sp*.* (21-22) *Cymbella* sp*.* (23) Unidentified diatom. (24-26) *Naiadinium* cf.*polonicum.* (27-29) *Carteria* spp. (30-31) *Tetraëdron minimum.* (32) *Cosmarium* sp. (33) *Staurastrum* sp. Scale bar = 20 μm.

**Supplementary Figure 3. FlowCam image library of *Dolichospermum* (=*Anabaena*) *flos*-*aquae* filaments**. Live samples. 10x objective, 100 μm flow cell. Scale bar = 20 μm. Heterocystes and akinetes, typical for *D. flos-aquae* can be seen.

**Supplementary Figure 4. Dot plots of flow cytometric sorting of phytoplankton populations from representative sites of Lake Burabay (A, B) and Lake Shchuchie (C, D), and representative images of sorted algae (E).** 1 – colonial picocyanobacteria, 2 – picophytoplankton; 3 – *Dinobryon* sp., 4 – centric diatoms, 5 – cryptomonads. Scale bar = 10 μm.

**Supplementary Figure 5. Comparison of picocyanobacteria (PCy) revealed by 16S next-generation sequencing in samples from Lake Burabay and Lake Shchuchie.**

**Legends to Supplementary Tables**

**Supplementary Table 1. Range of physico-chemical variables measured in June-September 2015 in Lakes Shchuchie and Burabay.**

**Supplementary Table 2. Main characteristics of Lakes Shchuchie and Burabay.**

**Supplementary Table 3. List of phytoplankton taxa in Lakes Shchuchie and Burabay identified by microscopy.** Phytoplankton community in Lake Shchuchie consisted of 167 species/subspecies from nine phyla – Bacillariophyta (71 species), Chlorophyta (35), Cyanobacteria (16), Ochrophyta (18), Miozoa (10), Cryptophyta (7), Charophyta (6), Euglenozoa (4), and Haptophyta (1). Phytoplankton in Lake Burabay composed of 243 species/subspecies from nine phyla, videlicet Bacillariophyta (87 species), Chlorophyta (54), Cyanobacteria (47), Ochrophyta (21), Miozoa (10), Cryptophyta (8), Euglenozoa (4), and Haptophyta (2).

**Supplementary Table 1.** **Range of physico-chemical variables measured in June-September 2015 in Lakes Shchuchie and Burabay.**

| Parameters | Shchuchie | | | |  | Burabay | | | |
| --- | --- | --- | --- | --- | --- | --- | --- | --- | --- |
|  | Mean | ±SD | Min | Max |  | Mean | ±SD | Min | Max |
| Water temperature, °C | 18.72 | 1.46 | 16.70 | 23.10 |  | 19.69 | 2.25 | 16.10 | 24.70 |
| Conductivity, mSm | 537.96 | 17.78 | 512 | 564 |  | 286.71 | 8.49 | 276 | 304 |
| pH | 8.54 | 0.12 | 8.32 | 8.92 |  | 8.77 | 0.22 | 8.32 | 9.12 |
| Dissolved Oxygen, % | 113.42 | 13.84 | 92 | 160 |  | 115.87 | 9.63 | 99 | 148 |
| Dissolved Oxygen, mg·L^-1^ | 10.02 | 1.11 | 7.80 | 13.00 |  | 10.11 | 1.08 | 7.20 | 12.10 |
| TDS, mg·L^-1^ | 274.38 | 16.44 | 257 | 320 |  | 183.42 | 5.36 | 177 | 194 |
| Light intensity, Klux | 59.37 | 24.78 | 12.00 | 90.10 |  | 50.87 | 26.09 | 12.58 | 86.10 |
| Secchi disk depth, m | 5.63 | 1.40 | 4.20 | 9.60 |  | 2.81 | 0.51 | 2.22 | 3.60 |
| Fluoride, mg·L^-1^ | 5.98 | 0.44 | 5.14 | 6.43 |  | 2.56 | 0.20 | 2.21 | 2.76 |
| Chloride, mg·L^-1^ | 34.69 | 7.16 | 31.93 | 63.40 |  | 13.27 | 1.02 | 11.31 | 14.76 |
| Bromide, mg·L^-1^ | 1.03 | 0.01 | 1.01 | 1.04 |  | 1.00 | 0.01 | 0.99 | 1.01 |
| Sulfate, mg·L^-1^ | 92.45 | 39.01 | 52.02 | 145.47 |  | 37.75 | 15.99 | 21.83 | 59.17 |
| Lithium, mg·L^-1^ | 0.05 | 0.001 | 0.05 | 0.05 |  | 0.03 | 0.00 | 0.03 | 0.03 |
| Sodium, mg·L^-1^ | 44.18 | 4.56 | 41.09 | 62.37 |  | 8.62 | 8.41 | 0.00 | 17.77 |
| Phosphate, mg·L^-1^ | 0.34 | 0.11 | 0.22 | 0.45 |  | 0.22 | 0.1 | 0.12 | 0.35 |
| Nitrate, mg·L^-1^ | 14.96 | 2.77 | 10.2 | 17.3 |  | 1.88 | 0.58 | 1.2 | 2.51 |
| Ammonium, mg·L^-1^ | 0.16 | 0.11 | 0.07 | 0.51 |  | 0.17 | 0.14 | 0.08 | 0.53 |
| Potassium, mg·L^-1^ | 6.65 | 0.60 | 6.07 | 8.04 |  | 4.11 | 0.51 | 3.82 | 5.53 |
| Calcium, mg·L^-1^ | 35.76 | 1.50 | 31.82 | 38.21 |  | 28.12 | 1.43 | 26.48 | 30.25 |
| Magnesium, mg·L^-1^ | 19.04 | 0.35 | 18.48 | 19.65 |  | 8.68 | 0.15 | 8.36 | 8.89 |

**Supplementary Table 2. Main characteristics of Lakes Shchuchie and Burabay** [52, 55].

| Lake | Coordinates | Maximum depth, m | Mean depth, m | Altitude, m | Watershed area, km^2^ | Surface area, km^2^ | Total P, μg L^-1^ |
| --- | --- | --- | --- | --- | --- | --- | --- |
| Shchuchie | 52°59' N 70°13' E | 22.7 | 11.2 | 391 | 64.4 | 14.9 | 8 |
| Burabay | 53°04' N 70°17' E | 6.5 | 3.4 | 315 | 164 | 10.2 | 16 |

**Supplementary Table 3. List of phytoplankton taxa in Lakes Shchuchie and Burabay identified by microscopy**. Phytoplankton community in Lake Shchuchie consisted of 167 species/subspecies from nine phyla – Bacillariophyta (71 species), Chlorophyta (35), Cyanobacteria (16), Ochrophyta (18), Miozoa (10), Cryptophyta (7), Charophyta (6), Euglenozoa (4), and Haptophyta (1). Phytoplankton in Lake Burabay composed of 243 species/subspecies from nine phyla, videlicet Bacillariophyta (87 species), Chlorophyta (54), Cyanobacteria (47), Ochrophyta (21), Miozoa (10), Cryptophyta (8), Euglenozoa (4), and Haptophyta (2).

| Species (*synonyms*) | FG | Shchuchie | | | | | Burabay | | | | |
| --- | --- | --- | --- | --- | --- | --- | --- | --- | --- | --- | --- |
|  |  | 2014 | 2015 | | | | 2014 | 2015 | | | |
|  |  | Aug. | June | July | Aug. | Sept. | Aug. | June | July | Aug. | Sept. |
| BACILLARIOPHYTA |  |  |  |  |  |  |  |  |  |  |  |
| *Achnanthidium eutrophilum* (Lange-Bertalot) Lange-Bertalot | MP |  | + | + |  |  |  |  |  |  |  |
| *Achnanthidium minutissimum* (Kützing) Czarnecki | MP | + | + |  | + | + | + | + | + | + | + |
| *Achnanthidium pyrenaicum* (Hustedt) H.Kobayasi (*=A. biasolettianum*) | MP |  |  |  |  |  |  |  | + |  |  |
| *Achnanthidium sieminskae* A.Witkowski, M.Kulikovskiy & C.Riaux-Gobin | MP |  |  |  |  |  |  |  |  | + | + |
| *Amphora* cf. *indistincta* Levkov | MP |  |  |  | + |  |  |  |  |  |  |
| *Amphora* cf. *pediculus* (Kützing) Grunow ex A.Schmidt | MP | + |  |  | + |  |  |  |  | + | + |
| *Amphora ovalis* (Kützing) Kützing | MP |  |  |  |  |  | + | + |  |  |  |
| *Aneumastus apiculatus* (Østrup) Lange-Bertalot (*=Navicula lacustris var. apiculata*) | MP |  |  |  | + |  |  |  |  |  |  |
| *Aneumastus minor* Lange-Bertalot (*=A. tusculus* var*. obtusus, Navicula tuscula* var*. obtusa, N. tuscula* f*. minor, N. tuscula* f*. obtusa*) | MP |  |  |  |  |  |  |  | + |  |  |
| *Aneumastus rostratus* (Hustedt) Lange-Bertalot (*=Navicula tuscula* var*. rostrata*) | MP |  |  |  | + |  |  |  |  |  |  |
| *Aneumastus stroesei* (Østrup) D.G.Mann (*=A. pseudotusculus, Navicula stroesei, N. pseudotuscula*) | MP | + |  |  | + |  |  |  |  |  |  |
| *Aneumastus tusculus* (Ehrenberg) Mann & Stickle (*=Navicula tuscula*) | MP | + | + | + | + |  | + |  | + | + |  |
| *Asterionella formosa* Hassall | C | + | + | + | + | + | + | + | + | + | + |
| *Aulacoseira ambigua* (Grunow) Simonsen | A |  |  |  |  |  | + | + | + | + | + |
| *Aulacoseira granulata* (Ehrenberg) Simonsen | P |  |  |  | + |  |  |  |  |  |  |
| *Brachysira microcephala* (Grunow) Compère (=*Navicula microcephala*) | MP |  |  |  |  |  |  | + |  | + |  |
| *Caloneis amphisbaena* (Bory) Cleve | MP |  |  |  | + |  |  |  |  |  |  |
| *Caloneis bacillum*  (Grunow) Cleve | MP |  |  |  | + |  |  |  |  |  |  |
| *Caloneis ventricosa* (Ehrenberg) F.Meister | MP |  |  | + |  |  |  |  |  |  |  |
| *Cocconeis placentula* Ehrenberg | MP |  | + |  |  |  | + |  |  | + | + |
| *Craticula* cf. *buderi* (Hustedt) Lange-Bertalot (*=Navicula pseudohalophila, N. buderi*) | MP | + |  |  |  |  |  |  |  |  |  |
| *Cyclotella radiosa* (Grunow) Lemmermann (*=C. comta* var*. radiosa, Puncticulata radiosa*) | A |  |  |  |  |  |  |  |  | + |  |
| *Cymbella affinis* Kützing | MP | + | + |  |  |  |  | + | + | + | + |
| *Cymbella helvetica* Kützing | MP |  |  |  |  |  |  |  |  | + |  |
| *Cymbella* cf. *cymbiformis* C.Agardh | MP | + |  |  | + |  | + |  | + |  |  |
| *Cymbella* cf. *lanceolata* (C.Agardh) C.Agardh | MP |  |  |  |  |  |  |  |  | + |  |
| *Cymbella neocistula* Krammer | MP | + |  |  | + | + | + | + | + | + | + |
| *Cymbopleura anglica* (Lagerstedt) Krammer (*=Cymbella anglica*) | MP |  |  |  |  |  |  |  |  | + |  |
| *Cymbopleura* cf. *amphicephala* (Nägeli) Krammer (*=Cymbella amphicephala*) | MP | + |  |  |  |  |  |  |  |  |  |
| *Cymbopleura* cf. *similiformis* K.Krammer | MP |  |  |  |  |  |  | + | + |  | + |
| *Cymbopleura lata* var. *truncata* Krammer | MP |  |  |  |  |  |  |  |  | + |  |
| *Diploneis* cf. *ovalis* (Hilse) Cleve | MP |  |  |  |  |  | + |  |  | + |  |
| *Encyonema caespitosum* Kützing | MP | + | + |  | + | + | + | + | + | + | + |
| *Encyonopsis subminuta* Krammer & E.Reichardt | MP |  |  |  |  |  |  | + |  |  |  |
| *Epithemia adnata* (Kützing) Brébisson | MP |  |  |  |  |  |  |  |  | + | + |
| *Epithemia* cf. *goeppertiana* Hilse | MP |  |  |  | + |  |  |  |  |  |  |
| *Epithemia sorex* Kützing | MP |  |  |  | + |  | + |  |  | + |  |
| *Fallacia* sp. | MP |  |  |  |  |  |  |  | + |  |  |
| *Fistulifera* sp*.* | MP |  |  |  |  | + |  |  |  |  |  |
| *Fragilaria amphicephaloides* Lange-Bertalot (*=F. amphicephala, F. capucina* var. *amphicephala*) | P |  |  |  | + |  |  |  |  | + | + |
| *Fragilaria capucina* Desmazières | P | + | + | + | + | + |  | + | + | + | + |
| *Fragilaria construens* (Ehrenberg) Grunow (*=Staurosira construens*) | MP | + |  |  | + | + | + | + | + | + |  |
| *Fragilaria crotonensis* Kitton | P | + |  | + | + | + | + | + |  |  | + |
| *Fragilaria vaucheriae* (Kützing) J.B.Petersen | P |  |  |  | + |  |  |  | + | + |  |
| *Gomphonema angustatum* (Kützing) Rabenhorst | MP |  |  |  |  |  | + | + | + |  |  |
| *Gomphonema* cf. *gracile* Ehrenberg | MP |  | + |  | + | + | + | + | + | + | + |
| *Gomphonema* cf. *kobayasii* Kociolek & J.C.Kingston | MP |  |  |  |  |  |  |  |  | + |  |
| *Gomphonema* cf. *parvulum* (Kützing) Kützing | MP |  | + | + |  |  |  |  |  |  |  |
| *Gomphonema pumilum* (Grunow) E.Reichardt & Lange-Bertalot (*= G. intricatum* var. *pumila, G. vibrio* var. *pumila*) | MP |  |  |  |  |  |  | + | + |  |  |
| *Gyrosigma* cf. *acuminatum* (Kützing) Rabenhorst | - | + |  |  |  |  |  |  |  |  |  |
| *Halamphora thumensis* (A.Mayer) Levkov (*=Amphora coffeiformis* var*. thumensis, A. thumensis*) | MP |  |  |  |  |  |  | + |  | + |  |
| *Hippodonta capitata* (Ehrenberg) Lange-Bertalot, Metzeltin & Witkowski (*=Navicula capitata, N. hungarica* var*. capitata*) | MP |  |  |  |  |  |  |  | + |  |  |
| *Hippodonta costulata* (Grunow) Lange-Bertalot, Metzeltin & Witkowski (*=Navicula costulata*) | MP |  |  |  | + |  |  |  |  | + |  |
| *Hippodonta costulatiformis* Lange-Bertalot, Metzeltin & Witkowski | MP |  |  |  |  |  |  |  |  | + |  |
| *Hippodonta hungarica* (Grunow) Lange-Bertalot, Metzeltin & Witkowski (*=Navicula hungarica*) | MP |  |  |  | + | + |  |  |  |  |  |
| *Hippodonta neglecta* Lange-Bertalot, Metzeltin & Witkowski | MP |  |  |  | + |  |  |  |  |  |  |
| *Karayevia* cf. *laterostrata* (Hustedt) Round & Bukhtiyarova (*=Achnanthes laterostrata*) | MP | + |  |  |  |  | + |  |  |  |  |
| *Karayevia clevei* (Grunow) Round & Bukhtiyarova (*=Achnanthes clevei, Achnanthidium clevei*) | MP |  |  |  | + |  |  |  |  | + | + |
| *Karayevia ploenensis* (Hustedt) Bukhtiyarova (*=Achnanthes ploenensis*) | MP |  |  |  |  |  |  |  |  | + |  |
| *Luticola* cf. *mutica* (Kützing) D.G.Mann | MP | + |  |  |  |  | + |  |  | + |  |
| *Mastogloia lacustris* (Grunow) Grunow | MP |  |  |  | + |  |  |  |  |  |  |
| *Mayamaea atomus* (Kützing) Lange-Bertalot | MP |  |  |  |  |  |  |  |  | + |  |
| *Meridion circulare* (Greville) C.Agardh | MP | + | + |  |  |  | + |  | + |  |  |
| *Navicula* cf. *antonii* Lange-Bertalot (*=N. menisculus* var*. grunowii*) | MP | + |  |  | + | + |  |  |  |  |  |
| *Navicula capitatoradiata* H.Germain | MP |  |  |  |  |  | + |  |  | + |  |
| *Navicula cryptocephala* Kützing | MP |  |  |  |  |  | + |  |  | + |  |
| *Navicula* cf. *erifuga* Lange-Bertalot | MP | + |  |  | + | + |  |  |  |  |  |
| *Navicula kohlmaieri* Lange-Bertalot | MP |  |  |  |  |  |  |  | + |  |  |
| *Navicula lanceolata* Ehrenberg | MP |  |  |  |  |  | + |  | + | + | + |
| *Navicula libonensis* Schoeman | MP |  |  |  | + |  |  |  | + |  |  |
| *Navicula* cf. *menisculus* Schumann | MP | + |  | + |  | + | + |  | + | + |  |
| *Navicula* cf. *notha* J.H.Wallace | MP |  |  |  |  |  |  | + |  |  |  |
| *Navicula oblonga* (Kützing) Kützing | MP |  |  |  | + |  |  |  |  | + |  |
| *Navicula* cf. *oligotraphenta* Lange-Bertalot & G.Hofmann | MP |  | + |  |  |  |  |  |  |  |  |
| *Navicula radiosa* Kützing | MP | + |  |  | + |  | + | + | + | + | + |
| *Navicula recens* (Lange-Bertalot) Lange-Bertalot | MP | + | + | + |  |  |  |  |  |  |  |
| *Navicula reichardtiana* Lange-Bertalot | MP |  |  |  | + |  |  |  |  |  |  |
| *Navicula slesvicensis* Grunow | MP |  |  |  |  |  |  |  |  | + |  |
| *Navicula subrostellata* Hustedt | MP |  |  |  | + |  |  |  |  |  |  |
| *Navicula trivialis* Lange-Bertalot | MP | + |  |  |  |  |  |  |  |  |  |
| *Navicula veneta* Kützing | MP |  |  |  |  |  | + |  |  | + | + |
| *Navicula weberi* Bahls | MP |  |  |  | + |  |  |  |  |  |  |
| *Neidiomorpha binodis* (Ehrenberg) M.Cantonati, Lange-Bertalot & N.Angeli (*=Navicula binodis*) | MP |  |  |  |  | + |  |  |  |  |  |
| *Nitzschia fonticola* (Grunow) Grunow (*=N. macedonica, N. palea* var*. fonticola*) | D |  |  |  |  |  |  |  |  | + |  |
| *Nitzschia inconspicua* Grunow | D |  |  |  |  |  |  |  | + |  | + |
| *Nitzschia* cf. *minuta* Bleisch | D |  |  |  |  |  |  |  |  | + |  |
| *Nitzschia* cf. *palea* (Kützing) W.Smith | D | + |  |  |  |  |  |  |  |  |  |
| *Nitzschia perminuta* (Grunow) M.Peragallo (*=N. frustulum* var*. perminuta, N. hiemalis*) | D |  |  |  | + | + |  |  |  |  |  |
| *Nitzschia recta* Hantzsch ex Rabenhorst | D |  |  |  |  |  | + |  | + | + |  |
| *Nitzschia vermicularis* (Kützing) Hantzsch in Rabenhorst | D |  |  |  |  |  |  | + |  |  |  |
| *Pantocsekiella comensis* (Grunow) K.T.Kiss & E.Ács (*=Cyclotella comensis, Lindavia comensis*) | A | + | + | + | + | + | + | + | + | + | + |
| *Pantocsekiella ocellata* (Pantocsek) K.T.Kiss & E.Ács (*=Cyclotella ocellata, Lindavia ocellata*) | B | + |  | + | + | + | + | + | + | + | + |
| *Pinnularia oriundiformis* Krammer | MP |  |  |  |  |  |  |  | + |  |  |
| *Pinnularia* cf. *viridis* (Nitzsch) Ehrenberg | MP |  |  |  |  |  | + |  |  |  |  |
| *Pinnularia saprophila* Lange-Bertalot, Kobayasi & Krammer | MP |  |  |  |  |  |  |  |  | + |  |
| *Placoneis elginensis* (W.Gregory) E.J.Cox (*=Navicula elginensis*) | MP |  |  |  |  | + | + |  |  | + |  |
| *Placoneis undulata* (Østrup) Lange-Bertalot (*=P. elginensis* var*. undulata, Navicula dicephala* var*. undulata*) | MP |  |  |  |  |  |  |  |  | + |  |
| *Plagiotropis* cf. *lepidoptera* (W.Gregory) Kuntze (*=Amphiprora lepidoptera, Tropidoneis lepidoptera*) | MP |  |  |  |  |  |  |  |  |  | + |
| *Planothidium biporomum* (M.H.Hohn & Hellerman) Lange-Bertalot (*=Achnanthes biporoma*) | MP |  |  |  |  |  |  | + | + |  |  |
| *Planothidium frequentissimum* (Lange-Bertalot) Lange-Bertalot (*=Achnanthes lanceolata* subsp*. frequentissima*) | MP |  |  |  |  |  |  |  | + |  | + |
| *Platessa conspicua* (Ant.Mayer) Lange-Bertalot (*=Achnanthes conspicua, Planothidium conspicuum*) | MP | + |  |  |  |  |  |  |  |  |  |
| *Pseudostaurosira elliptica* (Schumann) Edlund, Morales & Spaulding (*=Fragilaria elliptica*) | P |  |  |  |  |  |  |  | + | + |  |
| *Pseudostaurosiropsis connecticutensis* E.A.Morales | P |  |  |  |  |  |  |  | + |  |  |
| *Punctastriata linearis* D.M.Williams & Round | P |  |  |  | + |  |  | + |  | + |  |
| *Rhoicosphenia abbreviata* (C.Agardh) Lange-Bertalot | MP |  |  |  | + |  | + |  | + |  |  |
| *Rhopalodia* cf. *gibba* (Ehrenberg) Otto Müller | MP | + | + |  |  |  |  |  | + | + |  |
| *Sellaphora bacilloides* (Hustedt) Z.Levkov, S.Krstic & T.Nakov (*=Navicula bacilloides*) | MP |  |  |  | + |  |  |  |  | + |  |
| *Sellaphora* cf. *pupula* (Kützing) Mereschkovsky | MP | + |  |  |  |  | + | + |  | + |  |
| *Stauroneis* cf. *phoenicenteron* (Nitzsch) Ehrenberg | MP |  |  |  |  |  |  |  |  |  | + |
| *Staurosira venter* (Ehrenberg) Cleve & J.D.Möller (*=Fragilaria construens* var*. venter*) | P |  |  |  | + |  | + | + |  | + | + |
| *Staurosirella lapponica* (Grunow) D.M.Williams & Round (*=Fragilaria lapponica, Staurosira lapponica*) | P |  |  |  | + |  |  |  |  | + | + |
| *Staurosirella rhomboides* (Grunow) E.A.Morales & K.M.Manoylov (*=Fragilaria leptostauron* var*. rhomboids*) | P |  |  |  |  |  |  |  | + |  |  |
| *Stephanodiscus* sp. | B |  |  |  |  |  |  |  |  |  | + |
| *Surirella brebissonii* Krammer & Lange-Bertalot | MP |  |  |  |  |  | + |  |  | + |  |
| *Surirella* sp. | MP | + |  |  |  |  |  |  |  |  |  |
| *Tabellaria flocculosa* (Roth) Kützing | N |  |  |  |  |  |  |  |  | + |  |
| *Tryblionella angustata* W.Smith (*=Nitzschia angustata*) | D |  |  |  | + |  |  |  |  |  |  |
| *Ulnaria acus* (Kützing) Aboal (*=Synedra acus*) | D |  |  | + |  |  |  |  | + | + |  |
| *Ulnaria ulna* (Nitzsch) Compère (*=Synedra ulna, Fragilaria ulna*) | MP | + | + | + | + | + | + | + |  | + | + |
| OCHROPHYTA: CHRYSOPHYCEAE |  |  |  |  |  |  |  |  |  |  |  |
| *Bitrichia chodatii* (Reverdin) Chodat | X3 |  |  |  |  | + | + | + | + | + | + |
| *Chromulina* sp. | X3 |  |  |  |  |  |  |  |  | + |  |
| *Chrysococcus rufescens* G.A.Klebs | X3 | + | + |  |  |  | + | + | + |  | + |
| *Derepyxis* cf. *ollula* Stokes | - |  |  | + |  |  |  |  |  |  |  |
| *Dinobryon* cf. *elegans* Korshikov | E | + |  |  |  |  |  |  |  |  |  |
| *Dinobryon crenulatum* West & G.S.West | E |  |  | + |  |  |  | + |  |  |  |
| *Dinobryon divergens* O.E.Imhof | E | + |  | + | + |  | + | + | + | + | + |
| *Dinobryon sociale* (Ehrenberg) Ehrenberg | E | + | + | + | + |  | + | + | + | + | + |
| *Kephyrion boreale* Skuja | X2 |  |  |  |  |  |  | + |  |  |  |
| *Kephyrion littorale* J.W.G.Lund | X2 |  |  |  |  |  |  | + |  | + |  |
| *Kephyrion rubri-claustri* Conrad | X2 |  | + |  |  |  |  |  |  |  |  |
| *Kephyrion spirale* (Lackey) Conrad | X2 |  |  | + |  |  |  |  |  |  |  |
| *Ochromonas* sp. | X3 |  |  | + | + | + |  | + | + | + | + |
| *Pseudokephyrion conicum* Schiller (*=P. schilleri*) | X3 |  | + |  |  |  |  |  |  |  |  |
| *Pseudokephyrion entzii* W.Conrad | X3 | + | + | + | + |  |  |  |  |  |  |
| *Pseudokephyrion hyalinum* Hilliard | X3 |  |  |  |  |  |  | + |  |  |  |
| *Pseudokephyrion inflatum* Hilliard | X3 | + | + | + | + |  |  | + |  |  |  |
| *Pseudokephyrion minutissimum* Conrad | X3 |  | + |  |  |  |  |  |  |  |  |
| *Pseudokephyrion ovum* (Pascher & Ruttner) Conrad | X2 |  | + |  |  |  |  |  |  |  |  |
| OCHROPHYTA: SYNUROPHYCEAE |  |  |  |  |  |  |  |  |  |  |  |
| *Mallomonas crassisquama* (Asmund) Fott | E |  | + |  |  |  |  |  |  |  |  |
| *Mallomonas ploesslii* Perty (*=M. acaroides*) | E |  |  |  |  |  | + | + | + | + | + |
| *Mallomonas* sp. | E | + | + | + | + |  | + |  |  | + |  |
| *Mallomonas tonsurata* Teiling | E |  |  |  |  |  | + | + | + |  |  |
| *Spiniferomonas* sp. | X3 |  |  |  |  |  |  |  |  |  | + |
| OCHROPHYTA: XANTHOPHYCEAE |  |  |  |  |  |  |  |  |  |  |  |
| *Goniochloris* *contorta* (Bourrelly) Ettl | X2 |  |  |  |  |  | + |  |  |  |  |
| *Goniochloris* *sculpta* Geitler | X2 |  |  |  |  |  |  |  | + | + |  |
| *Tetraëdriella* cf. *regularis* (Kützing) Fott (*=Tetraëdron regulare*) | X1 |  |  |  |  |  |  |  |  | + |  |
| *Tetraëdriella jovetii* (Bourrelly) Bourrelly | X1 |  |  |  |  |  | + | + | + | + |  |
| OCHROPHYTA: RAPHIDOPHYCEAE |  |  |  |  |  |  |  |  |  |  |  |
| *Gonyostomum semen* (Ehrenberg) Diesing | Q |  |  |  |  |  | + |  |  |  |  |
| *Vacuolaria* cf. *virescens* Cienkowski | Q |  |  |  | + |  |  |  |  | + |  |
| CRYPTOPHYTA [Chromalveolata] |  |  |  |  |  |  |  |  |  |  |  |
| *Chroomonas* sp. | Y |  |  |  | + |  |  |  |  |  | + |
| *Cryptomonas curvata* Ehrenberg (*=C. rostrata, C.* *rostratiformis*) | Y | + | + | + | + |  | + | + |  | + |  |
| *Cryptomonas marssonii* Skuja | Y | + | + |  | + |  | + | + | + | + | + |
| *Cryptomonas ovata* Ehrenberg | Y | + | + | + | + | + | + | + | + | + | + |
| *Cryptomonas phaseolus* Skuja | Y | + | + | + | + |  | + | + | + | + |  |
| *Cryptomonas reflexa* (M.Marsson) Skuja | Y | + | + | + | + |  |  | + | + | + |  |
| *Plagioselmis lacustris* (Pascher & Ruttner) P.Javornicky (*=Rhodomonas lacustris*) | X2 |  |  |  |  |  | + | + | + | + | + |
| *Plagioselmis nannoplanctica* (H.Skuja) G.Novarino, I.A.N.Lucas & S.Morrall (*=Rhodomonas minuta* var*. nannoplanctica, R. lacustris* var*. nannoplanctica*) | X2 | + | + | + | + | + | + | + | + | + | + |
| HAPTOPHYTA [Chromalveolata] |  |  |  |  |  |  |  |  |  |  |  |
| *Chrysochromulina parva* Lackey | X2 | + | + | + | + | + | + | + | + | + | + |
| *Hymenomonas* *roseola* Stein | - |  |  |  |  |  |  |  |  | + |  |
| MIOZOA |  |  |  |  |  |  |  |  |  |  |  |
| *Ceratium hirundinella* (O.F.Müller) Dujardin | L_O_ | + | + | + | + | + | + | + | + | + | + |
| *Chimonodinium lomnickii* (Woloszynska) S.C. Craveiro, A.J.Calado, N.Daugbjerg, Gert Hansen & Ø.Moestrup (*=Peridinium lomnickii*) | W1 | + | + | + | + | + |  |  | + |  |  |
| *Gymnodinium lacustre* Schiller | L_O_ |  | + | + | + |  | + |  | + |  |  |
| *Gyrodinium helveticum* (Penard) Y.Takano & T.Horiguchi (*=Gymnodinium helveticum*) | L_O_ | + | + | + | + | + |  |  |  |  |  |
| *Naiadinium polonicum* (Woloszynska) S.Carty (*=Peridiniopsis* *polonicum, Peridinium polonicum*) | L_O_ | + | + |  |  |  | + |  |  |  |  |
| *Nusuttodinium aeruginosum* (F.Stein) Y.Takano & T.Horiguchi (*=Gymnodinium aeruginosum*) | L_O_ |  |  |  |  |  |  | + | + |  |  |
| *Parvodinium inconspicuum* (Lemmermann) S.Carty (*=Peridinium inconspicuum*) | L_O_ | + |  | + | + |  | + |  |  |  |  |
| *Parvodinium umbonatum* (Stein) S.Carty (*=Peridinium umbonatum*) | L_O_ |  |  |  |  |  |  |  | + |  |  |
| *Peridiniopsis elpatiewskyi* (Ostenfeld) Bourrelly (*=P. pygmaeum, Peridinium elpatiewskyi*) | L_O_ | + | + | + | + |  |  |  |  |  |  |
| *Peridiniopsis quadridens* (Stein) Bourrelly (*=Peridinium quadridens, Glenodinium quadridens*) | L_O_ | + |  |  | + |  |  |  |  |  |  |
| *Peridinium cinctum* (O.F.Müller) Ehrenberg | L_O_ | + |  | + | + | + | + |  | + | + | + |
| *Prosoaulax lacustris* (Stein) Calado & Moestrup (*=Amphidinium lacustre, A. elenkinii, A. lacustriforme, A. hyalinum*) | - | + |  | + | + |  |  | + |  |  |  |
| CHLOROPHYTA |  |  |  |  |  |  |  |  |  |  |  |
| *Acutodesmus* *acuminatus* (Lagerheim) P.M.Tsarenko (*=Scenedesmus acuminatus, Tetradesmus acuminatus*) | J |  |  |  |  |  | + |  |  |  |  |
| *Acutodesmus acutiformis* (Schröder) Tsarenko & D.M.John (*=Scenedesmus acutiformis, Enallax acutiformis*) | J | + |  |  |  |  | + | + |  |  |  |
| *Ankistrodesmus arcuatus* Korshikov (*=Monoraphidium arcuatum*) | X1 | + |  |  |  |  |  |  |  |  |  |
| *Ankyra judayi* (G.M.Smith) Fott | X1 |  |  |  |  |  | + |  |  |  |  |
| *Asterococcus limneticus* G.M.Smith | F |  |  | + |  |  |  |  |  |  |  |
| *Binuclearia lauterbornii* (Schmidle) Proschkina-Lavrenko (*=Planctonema lauterbornii*) | T |  |  |  |  |  | + | + | + |  | + |
| *Carteria* cf. *klebsii* (P.A.Dangeard) Francé | X1 | + |  |  |  |  |  |  |  |  |  |
| *Carteria* cf. *multifilis* (Fresenius) O.Dill | X1 |  |  | + |  |  |  |  |  |  |  |
| *Carteria* cf. *pascheri* Skuja | X1 | + |  |  |  |  |  |  |  |  |  |
| *Carteria pseudoglobosa* Ettl (*=C. globosa*) | X1 | + |  |  | + | + | + |  |  |  |  |
| *Carteria* sp. | X1 |  | + |  | + |  |  |  |  |  | + |
| *Chlamydomonas* cf. *debaryana* Goroschankin | X2 | + |  |  | + |  |  |  |  |  |  |
| *Chlamydomonas inaequalis* Pascher & Jahoda | X2 |  |  |  |  |  |  |  | + |  |  |
| *Chlamydomonas mutabilis* Gerloff | X2 |  |  |  |  |  |  |  |  | + |  |
| *Chlamydomonas* sp. | X2 | + |  |  | + |  |  | + |  | + | + |
| *Chlorella* cf. *vulgaris* Beyerinck | X1 |  | + |  |  |  |  |  |  |  |  |
| *Coelastrum astroideum* De Notaris | J |  |  |  |  |  |  | + |  |  |  |
| *Coenococcus* *planctonicus* Korshikov (*=Eutetramorus planctonicus*) | F |  |  |  |  |  | + |  |  |  |  |
| *Crucigeniella* cf. *rectangularis* (Nägeli) Komárek (*=Willea rectangularis,* *Crucigenia rectangularis*) | X1 |  |  |  |  |  |  | + |  |  |  |
| *Crucigeniella irregularis* (Wille) P.M.Tsarenko & D.M.John in D.M.John & P.M.Tsarenko (*=Willea irregularis,* *Crucigenia rectangularis* var*.* *irregularis, C. irregularis*) | X1 |  |  |  |  |  | + | + | + | + | + |
| *Desmodesmus abundans* (Kirchner) E.Hegewald (*=Scenedesmus abundans*) | J |  |  |  |  | + | + |  |  |  |  |
| *Desmodesmus armatus* (R.Chodat) E.Hegewald (*=Scenedesmus hystrix* var*. armatus*) | J | + |  |  |  |  |  |  |  | + |  |
| *Desmodesmus* *armatus* var. *bicaudatus* (Guglielmetti) E.Hegewald (*=Scenedesmus armatus* var*. bicaudatus*) | J |  |  |  |  |  |  |  |  | + |  |
| *Desmodesmus bicaudatus* (Dedusenko) P.M.Tsarenko (*=Scenedesmus bicaudatus*) | J |  |  | + |  | + |  |  |  |  |  |
| *Desmodesmus brasiliensis* (Bohlin) E.Hegewald (*=Scenedesmus brasiliensis*) | J |  |  |  |  |  |  |  |  | + |  |
| *Desmodesmus* cf. *microspina* (Chodat) Tsarenko (*=Scenedesmus microspina*) | J |  |  |  |  | + |  |  | + | + | + |
| *Desmodesmus pannonicus* (Hortobágyi) E.Hegewald (*=Scenedesmus pannonicus*) | J |  |  |  |  |  |  |  | + |  |  |
| *Desmodesmus perforatus* (Lemmermann) E.Hegewald (*=Scenedesmus perforatus*) | J |  |  | + |  |  |  |  |  |  |  |
| *Desmodesmus serratus* (Corda) S.S.An, Friedl & E.Hegewald (*=Scenedesmus serratus*) | J |  |  |  |  |  |  |  | + |  |  |
| *Didymocystis* cf. *inermis* (Fott) Fott | X1 |  |  |  |  |  |  |  |  |  | + |
| *Eudorina elegans* Ehrenberg | G |  |  |  |  |  |  |  |  | + |  |
| *Gloeotila scopulina* (Hazen) Heering (*=Klebsormidium scopulinum*) | T |  |  |  | + |  |  |  |  |  |  |
| *Golenkinia radiata* Chodat | J |  |  |  |  |  | + |  |  | + |  |
| *Hegewaldia parvula* (Woronichin) Pröschold, C.Bock, W.Luo & L Krienitz (*=Golenkiniopsis parvula*) | J |  |  |  |  |  | + |  |  |  |  |
| *Koliella* cf. *spirotaenia* (G.S.West) Hindák | X3 |  |  |  |  |  | + |  |  |  |  |
| *Lagerheimia genevensis* (Chodat) Chodat | X1 |  |  |  |  |  | + |  |  |  |  |
| *Lagerheimia wratislaviensis* Schröder | X1 |  |  |  |  |  | + |  |  |  |  |
| *Lemmermannia triangularis* (Chodat) C.Bock & Krienitz (*=Tetrastrum triangulare*) | J |  |  |  |  |  |  |  |  | + |  |
| *Monactinus simplex* (Meyen) Corda (*=Pediastrum simplex*) | J |  |  |  |  |  | + |  |  |  |  |
| *Monoraphidium griffithii* (Berkeley) Komárková-Legnerová (*=Ankistrodesmus acicularis*) | X1 |  |  |  |  | + | + | + | + | + |  |
| *Monoraphidium litorale* Hindák | X1 |  |  |  | + |  |  |  |  |  |  |
| *Monoraphidium minutum* (Nägeli) Komárková-Legnerová | X1 |  |  |  |  | + |  |  | + |  |  |
| *Mucidosphaerium pulchellum* (H.C.Wood) C.Bock, Proschold & Krienitz (*=Dictyosphaerium pulchellum*) | F |  |  |  | + |  |  |  |  |  |  |
| *Oocystis* cf. *pusilla* Hansgirg | F |  |  | + | + | + |  | + | + | + | + |
| *Oocystis lacustris* Chodat | F | + |  |  | + | + | + | + | + | + | + |
| *Oocystis rhomboidea* Fott | F |  |  |  |  |  |  | + |  |  |  |
| *Oocystis* sp. | F |  |  |  | + |  |  | + | + | + | + |
| *Pediastrum duplex* Meyen | J |  |  |  |  |  | + |  |  | + |  |
| *Phacotus lenticularis* (Ehrenberg) Deising | X_Ph_ |  |  |  | + |  |  |  |  |  |  |
| *Planktosphaeria gelatinosa* G.M.Smith | F |  |  | + | + | + |  | + | + | + | + |
| *Pseudodidymocystis planctonica* (Korshikov) E.Hegewald & Deason (*=Didymocystis planctonica*) | J | + |  |  | + | + | + |  |  |  | + |
| *Pseudopediastrum boryanum* (Turpin) E.Hegewald (*=Pediastrum boryanum*) | J |  |  |  | + |  | + |  |  | + |  |
| *Pseudopediastrum kawraiskyi* (Schmidle) E.Hegewald (*=Pediastrum kawraiskyi*) | J |  |  |  |  |  |  |  |  | + |  |
| *Quadrigula* cf. *closterioides* (Bohlin) Printz | F |  |  |  |  |  |  | + |  |  |  |
| *Quadrigula* cf. *pfitzeri* (Schröder) G.M.Smith | F |  |  |  |  |  | + |  |  | + |  |
| *Raphidocelis sigmoidea* Hindák | F |  |  |  |  |  | + |  |  |  |  |
| *Scenedesmus* cf. *apiculatus* Corda | J |  |  |  |  |  |  |  |  |  | + |
| *Scenedesmus ellipticus* Corda | J |  |  |  | + |  |  |  |  |  |  |
| *Scenedesmus quadricauda* (Turpin) Brébisson | J | + |  | + | + | + | + | + |  | + |  |
| *Sphaerocystis* cf. *planctonica* (Korshikov) Bourrelly | F | + |  |  | + |  |  | + | + | + | + |
| *Sphaerocystis* cf. *schroeteri* Chodat | F |  |  |  |  |  | + |  |  |  |  |
| *Stauridium tetras* (Ehrenberg) E.Hegewald | J |  |  |  |  |  | + |  |  |  | + |
| *Stichococcus bacillaris* Nägeli | X3 |  |  |  |  |  |  | + | + | + | + |
| *Tetrachlorella* *alternans* (G.M.Smith) Korshikov | X1 |  |  |  |  |  |  |  |  | + |  |
| *Tetradesmus* cf. *cumbricus* G.S.West | J |  |  |  |  |  |  |  |  | + |  |
| *Tetradesmus* *obliquus* (Turpin) M.J.Wynne (*=Scenedesmus acutus, S. obliquus, S. bijugatus, Acutodesmus obliquus*) | J |  |  | + | + | + | + |  | + | + | + |
| *Tetraëdron caudatum* (Corda) Hansgirg | J |  |  |  |  |  | + |  | + |  |  |
| *Tetraëdron minimum* (A.Braun) Hansgirg | J | + | + | + | + | + | + | + | + | + | + |
| *Tetraëdron triangulare* Korshikov | J |  | + |  |  |  |  |  |  |  |  |
| *Tetraselmis cordiformis* (H.J.Carter) Stein (*=Carteria cordiformis, Pyramichlamys cordiformis, Pteromonas angulosa* var*. cordiformis*) | X1 |  |  |  | + |  |  |  |  |  |  |
| CHAROPHYTA |  |  |  |  |  |  |  |  |  |  |  |
| *Closterium* cf. *acutum* Brébisson | P |  |  |  |  | + |  |  |  |  |  |
| *Closterium* cf. *gracile* Brébisson ex Ralfs | P |  |  |  |  |  | + |  |  |  |  |
| *Cosmarium abbreviatum* var. *minus* (West & G.S.West) Willi Krieger & Gerloff | N |  |  |  |  |  |  | + |  |  |  |
| *Cosmarium* cf. *bioculatum* Brébisson ex Ralfs | N |  |  |  |  |  |  |  | + |  |  |
| *Cosmarium baileyi* Wolle (*=C. depressum*) | N | + |  |  |  |  |  | + | + |  |  |
| *Cosmarium depressum* f. *minutum* Heimerl | N |  |  |  |  |  | + |  |  |  |  |
| *Cosmarium granatum* Brébisson ex Ralfs | N |  |  |  | + |  |  |  |  |  |  |
| *Cosmarium reniforme* (Ralfs) W.Archer | N |  |  |  |  |  | + |  |  |  |  |
| *Elakatothrix* cf. *gelatinosa* Wille | F |  |  |  |  |  |  |  | + |  | + |
| *Elakatothrix* *genevensis* (Reverdin) Hindák | F | + |  | + | + | + | + | + | + | + | + |
| *Mougeotia* sp. | T |  |  |  | + |  |  | + | + |  |  |
| *Spyrogyra* sp. | - |  |  |  |  |  |  |  |  | + |  |
| *Staurastrum* sp. | N |  |  |  | + |  |  |  |  |  |  |
| EUGLENOZOA |  |  |  |  |  |  |  |  |  |  |  |
| *Colacium* cf. *mucronatum* Bourrelly & Chadefaud | W1 |  |  |  |  |  |  |  |  | + |  |
| *Euglena* cf. *gracilis* Klebs | W1 |  | + | + |  |  |  |  |  |  |  |
| *Eutreptia* sp. | - | + |  |  | + |  |  |  |  |  |  |
| *Lepocinclis* sp. | W1 |  |  |  | + |  |  |  |  |  |  |
| *Trachelomonas* cf. *oblonga* Lemmermann | W2 |  |  |  |  |  |  | + |  |  |  |
| *Trachelomonas hispida* (Perty) F.Stein | W2 |  |  |  |  |  | + |  |  |  |  |
| *Trachelomonas volvocina* (Ehrenberg) Ehrenberg | W2 | + |  | + | + | + | + | + | + | + | + |
| CYANOBACTERIA |  |  |  |  |  |  |  |  |  |  |  |
| *Anathece bachmannii* (Komárek & Cronberg) Komárek, Kastovsky & Jezberová (*=Aphanothece bachmannii*) | K |  |  |  |  |  |  | + |  | + |  |
| *Anathece clathrata* (W.West & G.S.West) Komárek, Kastovsky & Jezberová (*=Aphanothece clathrata*) | K | + |  |  |  |  |  | + | + | + | + |
| *Anathece minutissima* (West) Komárek, Kastovsky & Jezberová (*=Aphanothece saxicola f. minutissima*) | K |  |  |  |  |  |  | + | + | + | + |
| *Anathece smithii* (Komárková-Legnerová & Cronberg) Komárek, Kastovsky & Jezberová (*=Aphanothece smithii*) | K |  |  |  |  |  |  |  | + | + |  |
| *Aphanocapsa* cf. *conferta* (West & G.S.West) Komárková-Legnerová & Cronberg (*=Microcystis pulverea* f. *conferta*) | K |  |  |  |  |  |  |  |  | + | + |
| *Aphanocapsa* cf. *planctonica* (G.M.Smith) Komárek & Anagnostidis (*=Microcystis pulverea* f. *planctonica*) | K |  |  | + |  |  | + |  | + | + | + |
| *Aphanocapsa* *delicatissima* West & G.S.West (*=Microcystis pulverea* f*. delicatissima, M. delicatissima*) | K |  |  | + |  |  | + | + | + |  |  |
| *Aphanocapsa elachista* West & G.S.West (*=Microcystis pulverea* f*. elachista, M. elachista*) | K |  |  |  |  |  | + |  | + | + | + |
| *Aphanocapsa holsatica* (Lemmermann) G.Cronberg & Komárek (*=Microcystis holsatica*) | K |  |  |  |  |  | + | + | + | + | + |
| *Aphanocapsa incerta* (Lemmermann) G.Cronberg & Komárek (*=Microcystis pulverea* f*. incerta, M. incerta*) | K |  |  |  |  |  | + |  |  | + | + |
| *Aphanocapsa parasitica* (Kützing) Komárek & Anagnostidis (*=Microcystis pulverea* f*. parasitica, M. parasitica*) | K |  |  |  |  |  | + |  |  |  |  |
| *Aphanocapsa* sp. | K |  |  |  |  |  |  | + | + |  |  |
| *Aphanothece nebulosa* Skuja | K |  |  |  |  |  |  |  | + |  | + |
| *Aphanothece* sp. | K |  |  |  |  |  |  |  |  | + |  |
| *Chroococcus aphanocapsoides* Skuja | L_O_ |  |  |  |  |  | + |  | + | + | + |
| *Chroococcus* cf. *minutus* (Kützing) Nägeli | L_O_ |  |  |  |  |  |  |  |  | + |  |
| *Chroococcus* *minimus* (Keissler) Lemmermann | L_O_ | + |  |  |  |  | + |  | + | + | + |
| *Chroococcus* sp. | L_O_ |  |  |  |  |  |  | + |  |  |  |
| *Cyanodictyon imperfectum* Cronberg & Weibull | K |  |  |  |  |  |  |  | + |  |  |
| *Cyanodictyon planctonicum* B.A.Mayer | K |  |  |  |  |  | + | + | + | + | + |
| *Cyanodictyon reticulatum* (Lemmermann) Geitler | K |  |  | + |  |  | + | + | + | + | + |
| *Cyanodictyon tubiforme* Cronberg | K |  |  |  |  |  | + | + | + | + | + |
| *Dolichospermum flos-aquae* (Brébisson ex Bornet & Flahault) P.Wacklin, L.Hoffmann & J.Komárek (*=Anabaena flos-aquae*) | H1 |  |  |  |  |  | + |  |  |  |  |
| *Dolichospermum mucosum* (Komárková-Legnerová & Eloranta) Wacklin, L.Hoffmann & Komárek (*=Anabaena mucosa*) | H1 |  |  |  |  |  |  |  |  | + | + |
| *Geitleribactron periphyticum* Komárek | MP |  |  |  |  |  | + | + | + | + |  |
| *Geitlerinema amphibium* (C.Agardh ex Gomont) Anagnostidis (*=Oscillatoria amphibia*) | S1 | + |  |  |  |  |  |  |  |  |  |
| *Geitlerinema splendidum* (Greville ex Gomont) Anagnostidis (*=Oscillatoria gracillima, O. leptotricha, O. splendida, Phormidium splendidum*) | S1 |  |  |  | + |  |  |  |  |  |  |
| *Gloeocapsa punctata* Nägeli | T_C_ |  |  |  |  |  |  |  |  |  | + |
| *Gomphosphaeria aponina* Kützing | L_O_ |  |  |  |  |  | + |  |  |  |  |
| *Leptolyngbya perelegans* (Lemmermann) Anagnostidis & Komárek (*=Lyngbya perelegans*) | T_C_ |  |  | + |  |  |  | + |  |  |  |
| *Limnococcus limneticus* (Lemmermann) Komárková, Jezberová, O.Komárek & Zapomelová (*=Chroococcus limneticus*) | L_O_ |  |  |  |  |  | + | + | + | + | + |
| *Limnothrix* cf. *guttulata* (Goor) I.Umezaki & M.Watanabe (*=Oscillatoria guttulata*) | S1 |  |  |  |  |  | + |  |  |  |  |
| *Limnothrix planctonica* (Woloszynska) Meffert (*=Oscillatoria planctonica*) | S1 | + |  |  |  |  |  |  |  | + |  |
| *Microcrocis* cf. *pulchella* (Buell) Geitler | MP |  |  |  |  |  | + |  |  |  |  |
| *Microcystis aeruginosa* (Kützing) Kützing | M |  |  |  |  |  | + |  |  | + |  |
| *Microcystis* cf. *smithii* Komárek & Anagnostidis (*=Aphanocapsa pulchra*) | M |  |  |  |  |  | + |  |  |  |  |
| *Microcystis wesenbergii* (Komárek) Komárek ex Komárek | L_M_ |  |  |  |  |  | + |  |  |  |  |
| *Oscillatoria tenuis* C.Agardh ex Gomont (*=Phormidium tenue, P. neotenue*) | MP | + |  |  |  |  |  |  |  |  |  |
| *Phormidium* sp. | T_C_ |  |  |  | + | + |  |  |  |  |  |
| *Planktolyngbya brevicellularis* G.Cronberg & Komárek | S1 |  |  |  |  |  |  |  |  | + | + |
| *Planktolyngbya limnetica* (Lemmermann) Komárková-Legnerová & Cronberg (*=Lyngbya limnetica, Oscillatoria splendida* var*. limnetica*) | S1 |  |  |  |  | + | + | + |  | + | + |
| *Planktothrix* sp. | S1 |  |  |  |  |  |  |  |  |  |  |
| *Pseudanabaena catenata* Lauterborn | MP |  |  |  |  |  | + |  | + |  |  |
| *Pseudanabaena galeata* Böcher | MP |  |  |  |  |  |  |  |  | + |  |
| *Pseudanabaena limnetica* (Lemmermann) Komárek (*=Oscillatoria limnetica*) | S1 | + |  |  | + |  | + | + |  |  |  |
| *Pseudanabaena mucicola* (Naumann & Huber-Pestalozzi) Schwabe (*=Phormidium mucicola*) | S1 | + |  |  |  |  |  | + |  |  |  |
| *Radiocystis geminata* Skuja | F |  |  |  |  |  |  |  |  | + |  |
| *Snowella lacustris* (Chodat) Komárek & Hindák (*=Gomphosphaeria lacustris*) | L_O_ |  |  |  |  |  | + |  |  | + |  |
| *Snowella litoralis* (Häyrén) Komárek & Hindák (*=Gomphosphaeria litoralis*) | L_O_ |  |  |  |  |  | + | + | + | + | + |
| *Synechococcus elongatus* (Nägeli) Nägeli | K |  |  |  |  |  |  |  |  | + |  |
| *Synechocystis minuscula* Woronichin | L_O_ |  |  |  |  |  | + |  | + |  | + |
| *Woronichinia compacta* (Lemmermann) Komárek & Hindák | L_O_ |  |  |  |  |  |  | + | + | + |  |

**FG** – functional groups sensu Reynolds *et al.* [85] with corrections by Padisák *et al.* [86]. Taxonomic position of eukaryotic algae is adjusted in accordance with Adl *et al.* [87, 88], and cyanobacteria in accordance with Komárek *et al.* [89]. Species names are verified with currently accepted names in AlgaeBase online database [90].


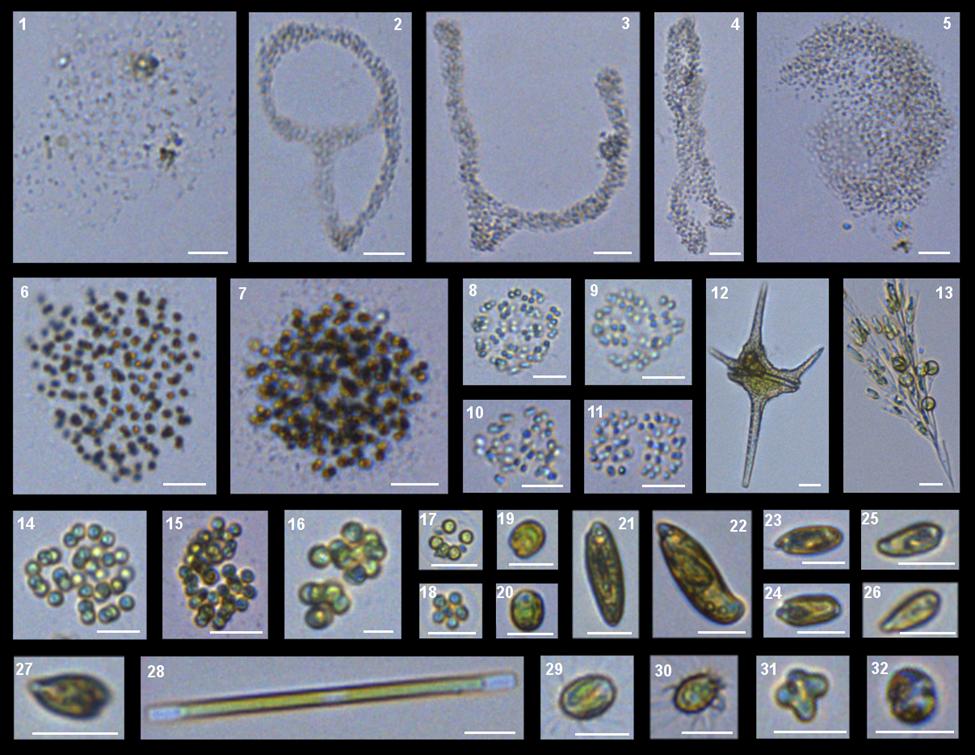


**Supplementary Figure 1**

**FlowCam image library of most common species in Lake Burabay.** Live samples. 10x objective, 100 μm flow cell. **(1)** *Cyanodictyon planctonicum.* **(2-4)** *Aphanocapsa holsatica.* **(5)** *Aphanocapsa elachista*. **(6)** *Aphanocapsa* cf. *planctonica.* **(7)** *Microcystis* cf. *smithii*. **(8-9)** *Snowella litoralis.* **(10)** *Aphanothece* sp. **(11)** *Chroococcus* *minimus.* **(12)** *Ceratium hirundinella*. **(13)** *Dinobryon sociale*. **(14-16)** *Coenococcus* cf*. planctonicus* **(17-18)** *Sphaerocystis* sp. **(19-20)** *Chlamydomonas* spp. **(21-22)** *Cryptomonas curvata*. **(23-24)** *Cryptomonas reflexa*. **(25-26)** *Plagioselmis nannoplanctica.*  **(27)** *Cryptomonas marssonii*. **(28)** *Ulnaria ulna*. **(29-30)** *Mallomonas* sp. **(31)** *Tetraëdriella jovetii.* **(32)** *Gymnodinium lacustre.* Scale bar = 20 μm.


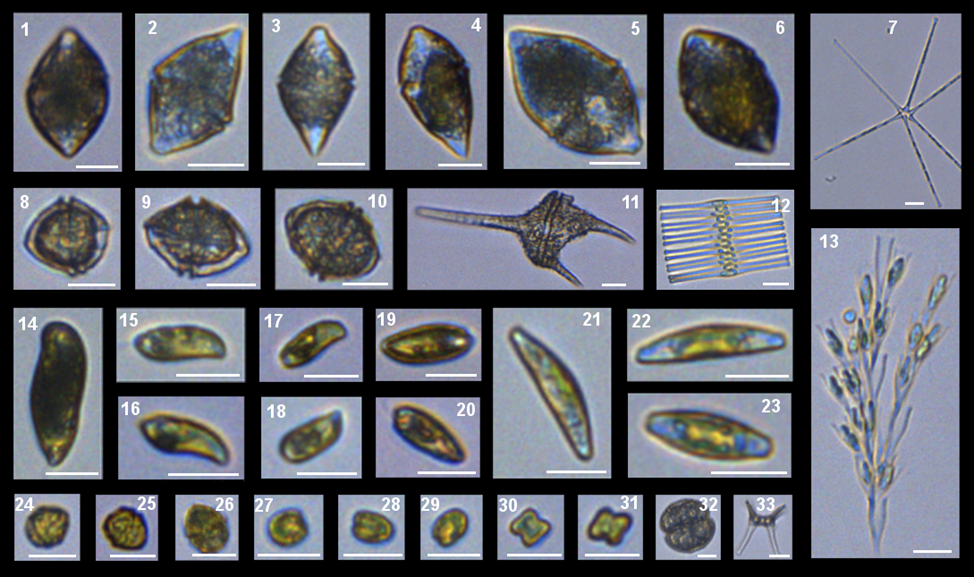


**Supplementary Figure 2.** **FlowCam image library of most common species in Lake Shchuchie.** Live samples. 10x objective, 100 μm flow cell. **(1-6)** *Gyrodinium helveticum.* **(7)** *Asterionella formosa*. **(8)** *Parvodinium* cf*. umbonatum* **(9-10)** *Peridiniopsis/Parvodinium* spp. **(11)** *Ceratium hirundinella*. **(12)** *Fragilaria crotonensis.* **(13)** *Dinobryon sociale.* **(14-17)** *Cryptomonas curvata.* **(18)** *Plagioselmis nannoplanctica.* **(19-20)** *Cryptomonas* sp*.* **(21-22)** *Cymbella* sp*.* **(23)** Unidentified diatom. **(24-26)** *Naiadinium* cf.*polonicum.* **(27-29)** *Carteria* spp. **(30-31)** *Tetraëdron minimum.* **(32)** *Cosmarium* sp. **(33)** *Staurastrum* sp. Scale bar = 20 μm.


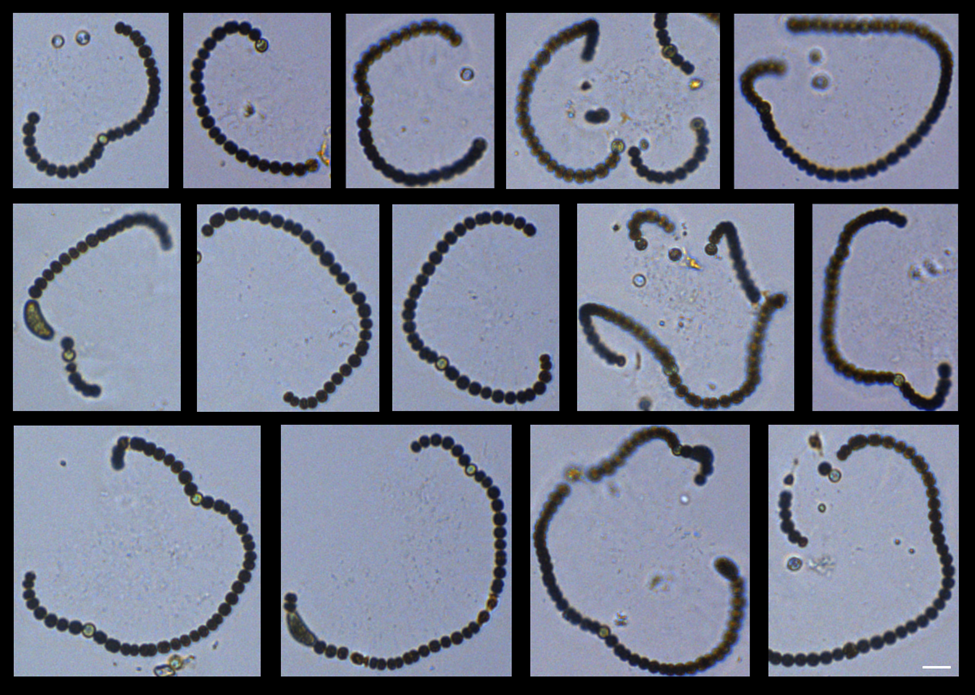


**Supplementary Figure 3**. **FlowCam image library of *Dolichospermum* (=*Anabaena*) *flos*-*aquae* filaments.** Live samples. 10x objective, 100 μm flow cell. Scale bar = 20 μm. Heterocystes and akinetes, typical for *D. flos-aquae* can be seen.


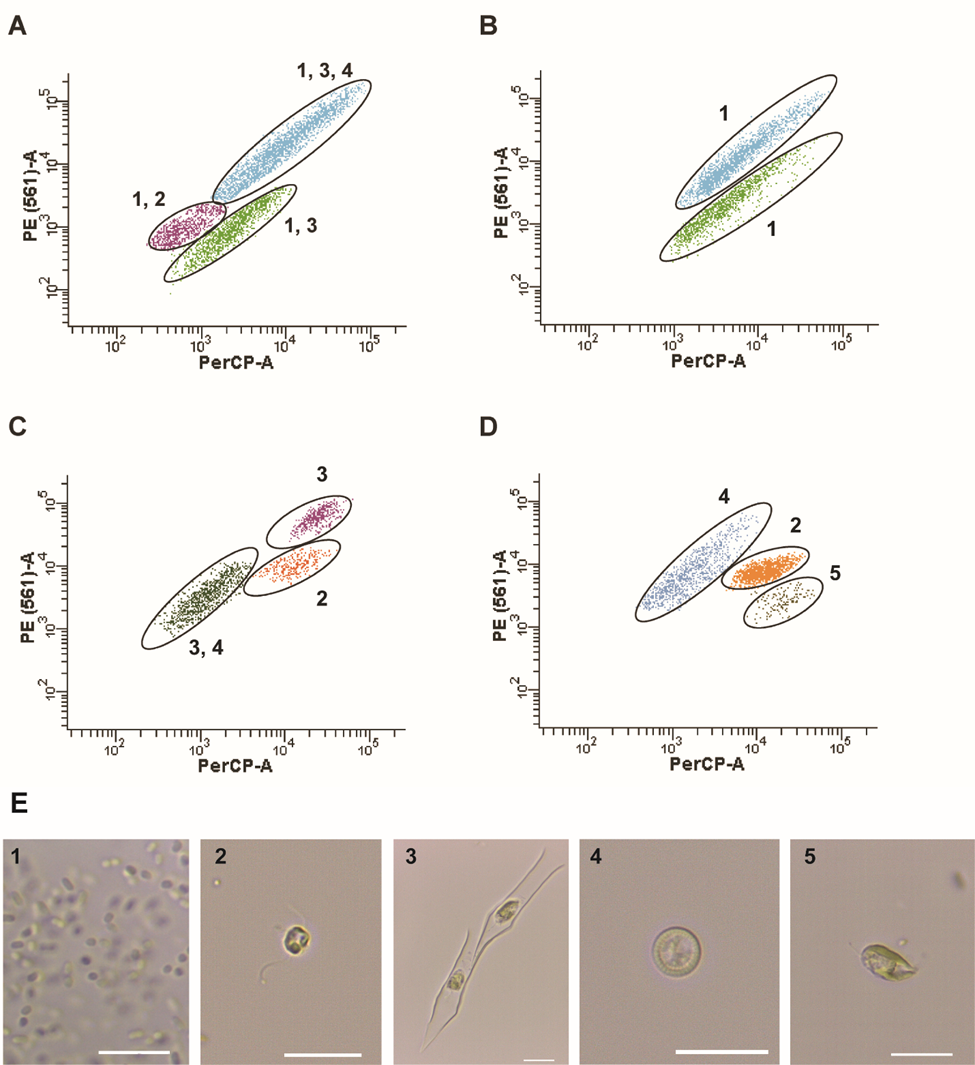


**Supplementary Figure 4.** **Dot plots of flow cytometric sorting of phytoplankton populations from representative sites of Lake Burabay (A, B) and Lake Shchuchie (C, D), and representative images of sorted algae (E).** 1 – colonial picocyanobacteria, 2 – picophytoplankton; 3 – *Dinobryon* sp., 4 – centric diatoms, 5 – cryptomonads. Scale bar = 10 μm.

**
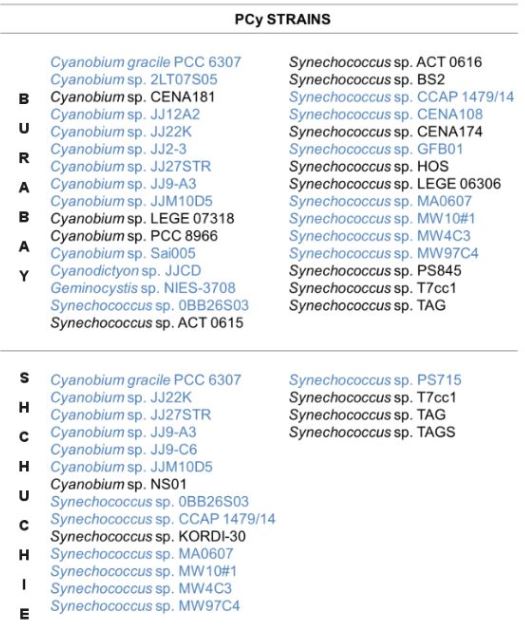
**

**Supplementary Figure 5.** A list of picocyanobacterial (PCy) strains in prokaryotic phytoplankton OTUs identified by NGS. Blue color highlights freshwater strains.

**Supplementary File 1.** **Results of statistical analysis of phytoplankton species distribution in Lakes Burabay and** **Shchuchie registered by NGS and microscopy**

**1. Spearman Rank Order Correlation**

**Data source:** Burabay, Microscopy / NGS full; Shchuchie, Microscopy / NGS full

Cell Contents:

Correlation Coefficient

P Value

Number of Samples

**Col Col 4 Col 5**

Col 1 0,824 0,943 0,783

0,0000002 0,0000002 0,000537

13 13 13

Col 2 0,883 0,895

0,0000002 0,0000002

13 13

Col 4 0,754

0,00214

13


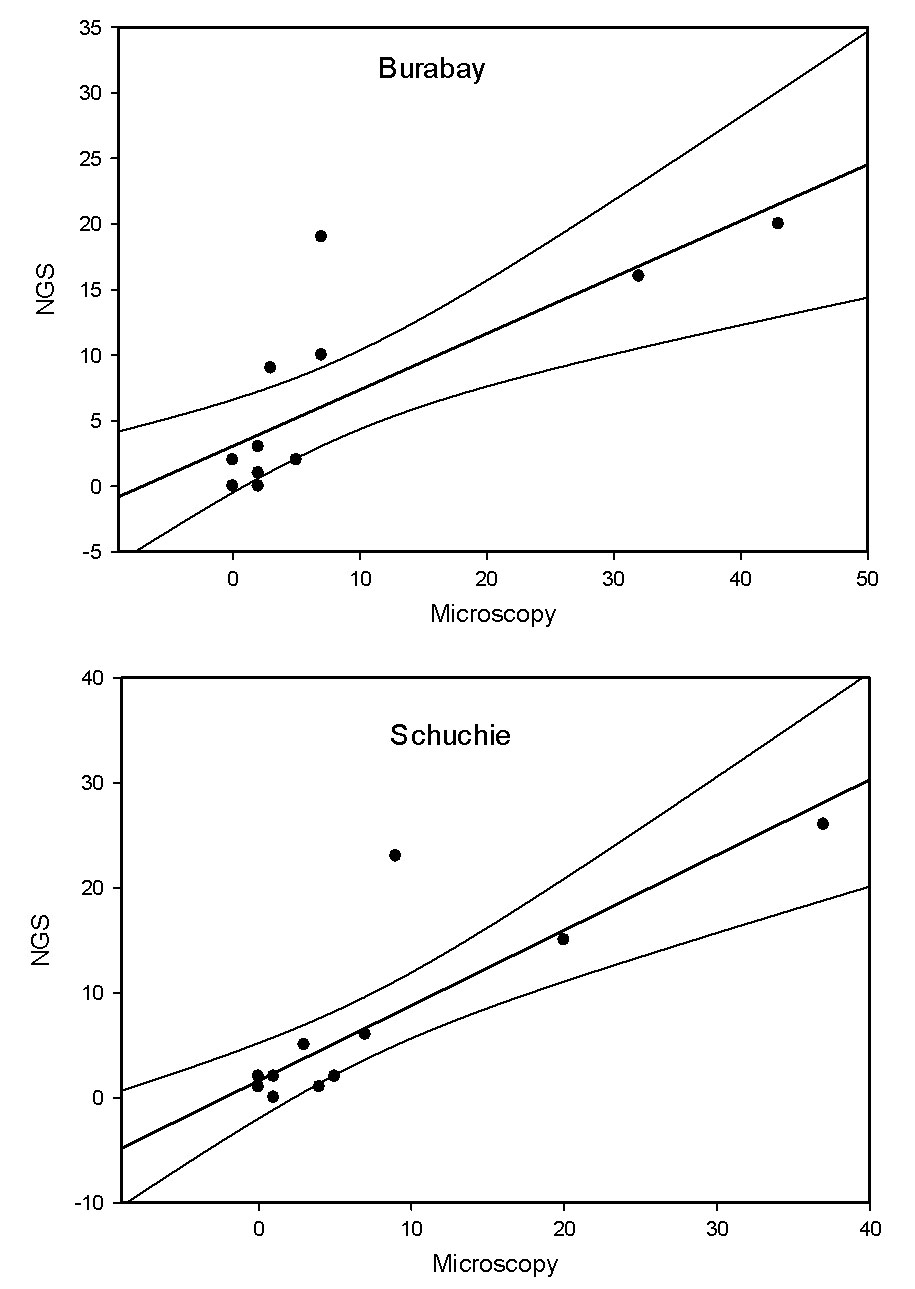


**2. Linear regression for Lake Burabay, Microscopy/NGS (14 groups):**

Coefficients (y=3.08+0.43*x):

b[0] 3,083

b[1] 0,429

r ² 0,601

path through origin (b=0): y=0.54*x

**3. Linear regression for Lake Shchuchie, Microscopy/NGS (14 groups):**

Coefficients (y=1.6+0.72*x):

b[0] 1,603

b[1] 0,717

r ² 0,725
